# Supplementary material for: The phylogenetic relationships of basal archosauromorphs, with an emphasis on the systematics of proterosuchian archosauriforms
Source: PeerJ. 2016 Apr 28;4:e1778. doi: 10.7717/peerj.1778 (PMC4860341; doi:10.7717/peerj.1778)
Supplement: Appendix S1 [file peerj-04-1778-s001.doc]

**Appendix 1**

Calculated ratios for the discretization of meristic characters.

Character 2:

*Chanaresuchus bonapartei* (PVL 4575): 0.53

*Dimorphodon* (Nesbitt, 2011: character 134): >0.50

*Euparkeria* (SAM-PK-5867): 0.38

*Heterodontosaurus* (SAM-PK-K1332): 0.34

*Macrocnemus bassanii* (PIMUZ T4355): ca. 0.22

*Mesosuchus* (SAM-PK-5882; Dilkes, 1998: fig. 23): 0.31

*Parasuchus hislopi* (Chatterjee, 1978: fig. 1: ISI R42): 0.72

*Petrolacosaurus* (Reisz, 1981: fig. 1): 0.24

*Prestosuchus chiniquensis* (UFRGS-PV-0629-T): ca. 0.34

*Prolacerta broomi* (Gow, 1975: fig. 27): 0.27

*Proterochampsa barrionuevoi* (PVSJ 606): 0.96

*Proterosuchus alexanderi* (NMQR 1484): ca. 0.34

*Protorosaurus* (BSPG 1995 I 5, cast of WMsN P 47361, skull length based on lower jaw length between its anterior tip and anterior border of the glenoid fossa): 0.34

*Pseudochampsa ischigualastensis* (PVSJ 567): 0.59

*Rhychosaurus articeps* (NHMUK PV R1237, R1238): ca. 0.32

*Silesaurus opolensis* (ZPAL AbIII/1930): 0.25

*Tropidosuchus romeri* (PVL 4601): ca. 0.44

*Turfanosuchus dabanensis* (IVPP V3237): ca. 0.29

*Vancleavea campi* (Nesbitt et al., 2009: fig. 18): ca. 0.28

*Youngina* (SAM-PK-K7710a): 0.36

Discretization by the cluster analysis: (1) 0.22−0.38; (2) 0.44−0.72; (3) 0.94−0.98. (5%=0.04). The first group is more inclusive than obtained in order to include several taxa with estimated ratio and proportionally small skulls.

Character 20:

*Azendohsaurus madagaskarensis* (Flynn et al., 2010: fig. 13a): 0.49

*Bentonyx* (BRSUG 27200): 0.33

*Boreopricea* (Tatarinov, 1978: 1b): 0.40

*Cerritosaurus binsfeldi* (CA s/n): 0.50

*Chanaresuchus bonapartei* (MCZ 4039): 0.54

*Chanaresuchus bonapartei* (MCZ 4037): 0.55

*Chanaresuchus bonapartei* (PVL 4586): 0.59

*Chanaresuchus bonapartei* (PVL 4575): 0.57

“*Chasmatosaurus*” *yuani* (IVPP V4067): 0.52

*Erythrosuchus africanus* (BP/1/5207): 0.52

*Dimorphodon* (Padian, 1983: fig. 5d): 0.72

*Euparkeria* (SAM-PK-5867): 0.47

*Fugusuchus* (Cheng, 1980: fig. 22): 0.43

*Garjainia prima* (PIN 2394/5): 0.55

*Gephyrosaurus* (Evans, 1980: fig. 1): 0.36

*Gracilisuchus* (MCZ 4117): 0.51

*Gualosuchus reigi* (PULR 05): 0.57

*Gualosuchus reigi* (PVL 4576): 0.55

*Herrerasaurus* (PVSJ 407): 0.57

*Heterodontosaurus* (AM unnumbered): 0.39

*Heterodontosaurus* (SAM-PK-K337): 0.38

*Heterodontosaurus* (SAM-PK-K1332): 0.37

*Jesairosaurus lehmani* (ZAR 06): ca. 0.47

*Macrocnemus bessanii* (PIMUZ T4822): 0.56

*Mesosuchus* (Dilkes, 1998: fig. 2c): 0.29

*Nicrosaurus kapffi* (SMNS 4379: Hungerbühler, 1998: table 2.2.1): 0.73

*Nicrosaurus kapffi* (SMNS 4378: Hungerbühler, 1998: table 2.2.1): 0.70

*Nicrosaurus kapffi* (SMNS 5727: Hungerbühler, 1998: table 2.2.1): 0.70

*Nicrosaurus kapffi* (SMNS 5726: Hungerbühler, 1998: table 2.2.1): 0.72

*Ornithosuchus* (Sereno, 1991: fig. 11a): 0.56

*Parasuchus hislopi* (ISIR 42): 0.76

*Petrolacosaurus* (Reisz, 1982: fig. 2): 0.35

*Planocephalosaurus* (Fraser, 1981: fig. 1a): 0.35

*Prestosuchus chiniquensis* (UFRGS-PV-0156-T): 0.56

*Prolacerta* (BP/1/471): 0.45

*Prolacerta* (SAM-PK-K10797): 0.45

*Proterochampsa barrionuevoi* (Dilkes & Arcucci, 2012: fig. 4): 0.59

*Proterochampsa barrionuevoi* (PVL 2063): 0.62

*Proterochampsa barrionuevoi* (PVSJ 606): 0.58

*Proterochampsa nodosa* (MCP 1694-Pv): 0.61

*Proterosuchus goweri* (NMQR 880): 0.51

*Proterosuchus fergusi* (RC 846): 0.50

*Proterosuchus fergusi* (SAM-PK-11208): 0.51

*Protorosaurus* (USNM 442453, cast of NMK S 180): 0.56

*Pseudochampsa ischigualastensis* (PVSJ 567): 0.50

*Rhynchosaurus articeps* (SHYMS 3): ca. 0.34

*Riojasuchus tenuisceps* (PVL 3827): 0.50

*Shansisuchus* (Wang et al., 2013: fig. 2c): 0.49

*Simoedosaurus lemoinei* (Russell-Sigogneau & Russell, 1978: fig. 1): 0.39

*Smilosuchus gregorii* (UCMP 27200): 0.70

*Smilosuchus adamanensis* (Camp, 1930: fig. 11a): 0.72

*Smilosuchus lithodendrorum* (Camp, 1930: fig. 11c): 0.70

*Tanystropheus longobardicus* (Wild, 1973: fig. 10b): 0.52

*Tropidosuchus romeri* (PVL 4601): 0.49

*Turfanosuchus dabanensis* (IVPP V3237): ca. 0.50

*Vancleavea campi* (USNM 508579 cast of GR 138): 0.30−0.34

*Youngina* (GHG K 106): 0.49

*Youngosuchus sinensis* (IVPP V3239): 0.59

Discretization by the cluster analysis: (1) 0.29−0.40, (2) 0.43−0.62; (3) 0.70−0.76. (5%=0.02).

Character 21:

*Azendohsaurus madagaskarensis* (Flynn et al., 2010: fig. 13a): 0.62

*Batrachotomus kupferzellensis* (Gower, 1999: fig. 2a): 0.78

*Bentonyx* (BRSUG 27200): 0.49

*Boreopricea* (Tatarinov, 1978: 1b): 0.48

*Cerritosaurus binsfeldi* (CA s/n): 0.39

*Chanaresuchus bonapartei* (MCZ 4037): 0.27

*Chanaresuchus bonapartei* (MCZ 4039): <0.22

*Chanaresuchus bonapartei* (PVL 4575): 0.15

*Chanaresuchus bonapartei* (PVL 4586): 0.21

“*Chasmatosaurus*” *yuani* (IVPP V4067): ca. 0.42

*Dimorphodon* (Padian, 1983: fig. 5d): 0.63

*Erythrosuchus* (BP/1/5207): 0.52

*Euparkeria* (SAM-PK-5867): ca. 0.71

*Garjainia prima* (PIN 2394/5): 0.70

*Gephyrosaurus* (Evans, 1980: fig. 1): 0.30

*Gracilisuchus* (MCZ 4117): ca. 0.54

*Gualosuchus reigi* (PULR 06): 0.26

*Gualosuchus reigi* (PVL 4576): 0.22

*Herrerasaurus* (PVSJ 407): 0.65

*Heterodontosaurus* (SAM-PK-K1332): 0.61

*Macrocnemus bessanii* (PIMUZ T4822): ca. 0.28

*Mesosuchus* (SAM-PK-6536): 0.50

*Nicrosaurus kapffi* (SMNS 4379: Hungerbühler, 1998: fig. 2.10): 1.04

*Ornithosuchus* (Sereno, 1991: fig. 11a): 0.52

*Parasuchus hislopi* (ISIR 42): 0.39

*Petrolacosaurus* (Reisz, 1981: fig. 2): 0.44

*Planocephalosaurus* (Fraser, 1982: fig. 1a): 0.45

*Prestosuchus chiniquensis* (UFRGS-PV-0156-T): 0.69

*Prolacerta* (BP/1/471): 0.42

*Proterochampsa barrionuevoi* (Dilkes & Arcucci, 2012: fig. 5a, b): 0.38

*Proterochampsa barrionuevoi* (PVL 2063): 0.41

*Proterochampsa nodosa* (MCP 1694-Pv): 0.59

*Proterosuchus fergusi* (RC 846): 0.39

*Proterosuchus goweri* (NMQR 880): <0.44

*Rhynchosaurus articeps* (SHYMS 1): ca. 0.44

*Riojasuchus tenuisceps* (PVL 3827): 0.73

*Shansisuchus* (Wang et al., 2013: fig. 2b): 0.59

*Smilosuchus gregorii* (UCMP 27200): 0.65

*Smilosuchus adamanensis* (Camp, 1930: fig. 11a): 0.72

*Smilosuchus lithodendrorum* (Camp, 1930: fig. 11c): 0.74

*Trilophosaurus buettneri* (Spielmann et al., 2008: fig. 20a, TMM 31025-207): 0.80

*Turfanosuchus dabanensis* (IVPP V3237): ca. 0.57

*Vancleavea campi* (USNM 508579 cast of GR 138): ca. 0.50

*Youngosuchus sinensis* (IVPP V3239): 0.53

Discretization by the cluster analysis: (1) 0.15−0.30; (2) 0.38−0.53; (3) 0.59−0.80; (4) 1.04. (5%=0.04).

Character 28:

*Aetosauroides scagliai* (PVL 2059): >3.27

*Archosaurus* holotype (PIN 1100/55): 3.72

*Asperoris* (NHMUK PV R36615): 1.26

*Azendohsaurus madagaskarensis* (UA 8-7-98-284): 1.40

*Azendohsaurus madagaskarensis* (FMNH PR 2751): 1.25

*Batrachotomus kupferzellensis* (SMNS 52970): 1.51

*Batrachotomus kupferzellensis* (SMNS 80260): 1.83

*Boreopricea* (Tatarinov, 1978: 1b): 2.73

*Cerritosaurus binsfeldi* (CA s/n): ca. 1.50

*Chanaresuchus bonapartei* (MCZ 4039): 3.18

*Chanaresuchus bonapartei* (MCZ 4037): 3.19

*Chanaresuchus bonapartei* (PVL 4586): 4.15

*Chanaresuchus bonapartei* (PVL 4575): 3.27

“*Chasmatosaurus*” *yuani* (IVPP V4067): 3.49

“*Chasmatosaurus*” *yuani* (IVPP V90002): 4.37

*Chasmatosuchus vjushkovi* (PIN 2394/4): 2.78

*Cteniogenys* sp. (NHMUK PV R11730): >3.91

*Dimorphodon* (NHMUK PV R41212-13): 4.68

*Erythrosuchus* (BP/1/5207): 1.50

*Erythrosuchus* (BP/1/4526): 1.65

*Euparkeria* (UMZC T692): 1.24

*Euparkeria* (SAM-PK-6047a): 1.24

*Euparkeria* (SAM-PK-13665): 1.66

*Garjainia madiba* (BP/1/6232N): 2.22

*Garjainia madiba* (BP/1/6232L): 2.83

*Garjainia prima* (PIN 2394/5): 1.82

*Gephyrosaurus* (Evans, 1980: fig. 29): 1.49

*Gracilisuchus* (MCZ 4117): 1.93

*Gualosuchus reigi* (PULR 05): 3.40

*Gualosuchus reigi* (PVL 4576): 3.22

*Herrerasaurus* (PVSJ 407): 1.22

*Heterodontosaurus* (AM unnumbered): 1.86

*Heterodontosaurus* (SAM-PK-K337): 1.49

*Heterodontosaurus* (SAM-PK-K1332): 1.91

*Macrocnemus bessanii* (PIMUZ T4822): 3.24

*Mesosuchus* (Dilkes, 1998: fig. 7a): 1.39

*Nicrosaurus kapffi* (SMNS 4379: Hungerbühler, 1998: fig. 2.10): 2.46

*Ornithosuchus* (NHMUK PV R2409): 3.20

*Parasuchus hislopi* (ISIR 42): 8.89

*Petrolacosaurus* (Reisz, 1981: fig. 2): 3.00

*Planocephalosaurus* (Fraser, 1982: plate 70, fig. 1): 2.83

*Prestosuchus chiniquensis* (UFRGS-PV-0156-T): 0.71

*Prestosuchus chiniquensis* (UFRGS-PV-0152-T): 1.14

*Prolacerta* (BP/1/471): 3.80

*Prolacerta* (BP/1/4504): 2.93

*Proterochampsa barrionuevoi* (Dilkes & Arcucci, 2012: fig. 5a, b): 2.33

*Proterosuchus fergusi* (RC 59): 3.50

*Proterosuchus fergusi* (SAM-PK-K140): 2.39

*Proterosuchus fergusi* (SAM-PK-11208): 3.19

*Proterosuchus fergusi* (BP/1/3993): 3.03

*Proterosuchus fergusi* (TM 201): 3.03

*Proterosuchus goweri* (NMQR 880): ca. 3.64

*Protorosaurus* (USNM 442453, cast of NMK S 180): 2.59

*Pseudochampsa ischigualastensis* (PVSJ 567): 3.10

*Riojasuchus tenuisceps* (PVL 3827): 2.50

*Sarmatosuchus* (PIN 2865/68): 2.29

*Shansisuchus shansisuchus* (Young, 1964: figs. 8, 9): 1.07−1.33

*Shansisuchus shansisuchus* (Wang et al., 2013: fig. 2c): 1.30

*Shansisuchus kuyeheensis* (Cheng, 1980: fig. 24): ca. 1.57

*Silesaurus opolensis* (ZPAL AbIII/361/34): 2.00

*Smilosuchus gregorii* (UCMP 27200): 3.24

*Smilosuchus adamanensis* (Camp, 1930: fig. 11a): 4.32

*Smilosuchus lithodendrorum* (Camp, 1930: fig. 11c): 3.00

*Tanystropheus longobardicus* (Wild, 1973: fig. 10b): 2.44

*Trilophosaurus buettneri* (Spielmann et al., 2008: fig. 20a, TMM 31025-207): 2.58

*Turfanosuchus dabanensis* (IVPP V3237): 1.69

*Vancleavea campi* (USNM 508579 cast of GR 138): 1.75

*Youngina* (SAM-PK-K7578): 2.00

*Youngosuchus sinensis* (IVPP V3239): 1.44

Discretization by the cluster analysis: (1) 0.70−0.73; (2) 1.07−2.00; (3) 2.22−3.80; (4) 4.15−4.68. (5%=0.20).

Character 50:

*Aetosauroides scagliai* (PVL 2059): <0.62

*Batrachotomus kupferzellensis* (SMNS 52970): <0.41

*Cerritosaurus binsfeldi* (CA s/n): 0.76

*Chalishevia* (PIN 4366/1): <0.47

*Chanaresuchus bonapartei* (MCZ 4039): 0.49

*Chanaresuchus bonapartei* (MCZ 4037): 0.65

*Chanaresuchus bonapartei* (PVL 4586): 0.51

*Chanaresuchus bonapartei* (PVL 4575): 0.47

“*Chasmatosaurus*” *yuani* (IVPP V4067): 0.36

*Dimorphodon* (NHMUK PV R41212-13): 0.54

*Erythrosuchus* (BP/1/5207): 0.43

*Euparkeria* (SAM-PK-5867): 0.22

*Euparkeria* (SAM-PK-6047a): 0.17

*Euparkeria* (SAM-PK-13665): 0.16

*Fugusuchus* (GMB V313 unpublished picture): >0.33

*Garjainia prima* (PIN 2394/5): 0.35

*Gracilisuchus* (MCZ 4117): 0.12

*Gualosuchus reigi* (PULR 05): 0.53

*Gualosuchus reigi* (PVL 4576): 0.58

*Guchengosuchus* (IVPP V8808-1): 0.36

*Herrerasaurus* (PVSJ 407): 0.42

*Heterodontosaurus* (SAM-PK-K1332): 0.47

*Jaxtasuchus salomoni* (SMNS 91083): 0.60

*Kalisuchus* (QM F8998): <0.37

*Lewisuchus* (PULR 01): <0.35

*Marasuchus lilloensis* (PVL 3870): <0.41

*Nicrosaurus kapffi* (SMNS 4379: Hungerbühler, 1998: fig. 2.10): 0.56

*Ornithosuchus* (Sereno, 1991: fig. 11a): 0.35

*Parasuchus hislopi* (ISIR 42): 0.64

*Prestosuchus chiniquensis* (UFRGS-PV-0152-T): 0.38

*Prestosuchus chiniquensis* (UFRGS-PV-0156-T): 0.43

*Proterochampsa barrionuevoi* (Dilkes & Arcucci, 2012: figs. 4, 5): 0.73

*Proterosuchus alexanderi* (NMQR 1484): 0.43

*Proterosuchus fergusi* (BP/1/3993): 0.39

*Proterosuchus fergusi* (BSPG 1934 VIII 514): 0.33

*Proterosuchus fergusi* (GHG 231): 0.32

*Proterosuchus fergusi* (RC 59): 0.34

*Proterosuchus fergusi* (RC 846): 0.36

*Proterosuchus fergusi* (SAM-PK-11208): 0.35

*Proterosuchus fergusi* (SAM-PK-K140): 0.36

*Proterosuchus goweri* (NMQR 880): 0.31

*Pseudochampsa ischigualastensis* (PVSJ 567): 0.67

*Rhadinosuchus gracilis* (BSPG AS XXV 50): 0.52

*Riojasuchus tenuisceps* (PVL 3827): 0.39

*Shansisuchus shansisuchus* (Wang et al., 2013: fig. 2a): 0.36

*Silesaurus opolensis* (ZPAL AbIII/361/26, 1218): ca. 0.29

*Smilosuchus gregorii* (UCMP 27200): 0.54

*Smilosuchus adamanensis* (Camp, 1930: fig. 11a): 0.47

*Smilosuchus lithodendrorum* (Camp, 1930: fig. 11c): 0.48

*Tasmaniosaurus* (UTGD 54655): >0.29

*Tropidosuchus romeri* (PVL 4606): 0.39

*Turfanosuchus dabanensis* (IVPP V3237): ca. 0.19

*Youngosuchus sinensis* (IVPP V3239): 0.34

Discretization by the cluster analysis: (1) 0.12−0.22; (2) 0.29−0.60; (3) 0.64−0.76. (5%=0.03).

Character 55:

*Aetosauroides scagliai* (PVL 2052): 0.21

*Aetosauroides scagliai* (PVL 2059): 0.18

*Batrachotomus kupferzellensis* (Gower, 1999: fig. 2a): 0.17

*Cerritosaurus binsfeldi* (CA s/n): 0.28

*Dimorphodon* (NHMUK PV R41212-13): 0.11

*Erythrosuchus* (BP/1/5207): 0.11

*Euparkeria* (SAM-PK-6047a): 0.10

*Gracilisuchus* (MCZ 4117): 0.23

*Herrerasaurus* (PVSJ 407): 0.09

*Heterodontosaurus* (SAM-PK-K1332): 3.00

*Ornithosuchus* (Sereno, 1991: fig. 11a): 0.43

*Parasuchus angustifrons* (BSPG 1931 X 502): 0.18

*Parasuchus hislopi* (ISIR 42): 0.23

*Prestosuchus chiniquensis* (UFRGS-PV-0156-T): 0.14

*Proterochampsa barrionuevoi* (Dilkes & Arcucci, 2012: figs. 4): 0.90−0.94

*Riojasuchus tenuisceps* (PVL 3827): 0.31

*Shansisuchus shansisuchus* (Wang et al., 2013: fig. 2a): 0.18

*Tropidosuchus romeri* (PVL 4601): 0.18

*Turfanosuchus dabanensis* (IVPP V3237): 0.12

Discretization by the cluster analysis: (1) 0.09−0.23; (2) 0.28−0.43; (3) 0.90−0.94. (5%=0.04). *Heterodontosaurus* was excluded from the cluster analysis because of a strongly reduced antorbital fenestra.

Character 75:

*Acerosodontosaurus* (Bickelmann, Müller & Reisz, 2009; MNHN 1908-32-57): > 36

*Aetosauroides scagliai* (PVL 2059): ≥10

*Amotosaurus* (SMNS unnumbered): 25

*Asperoris* (NHMUK PV R36615; Nesbitt, Butler & Gower, 2013: 8): ≥10

*Azendohsaurus madagaskarensis* (Flynn et al., 2010: 676): 14

*Batrachotomus kupferzellensis* (Gower, 1999: 15): 11

*Boreopricea* (Tatarinov, 1978: 510): 33

*Cerritosaurus binsfeldi* (CA s/n): 10−14

*Chalishevia* (PIN 4366/1): 12−13

*Chanaresuchus bonapartei* (Romer, 1971a: 13): 18

“*Chasmatosaurus ultimus*” (IVPP V2301): 13−14

“*Chasmatosaurus*” *yuani* (IVPP V2719): 29

*Dimorphodon* (NHMUK PV R41212-13): 8

*Doswellia kaltenbachi* (USNM 186989, based on the dentary tooth count): >25

*Erythrosuchus* (BP/1/5207): 11

*Euparkeria* (Ewer, 1965): 13

*Fugusuchus* (Cheng, 1980: fig. 22): 17−18

*Garjainia prima* (PIN 2394/5): 13−14

*Gephyrosaurus* (Evans, 1980: 225): 40

GHG 7433MI: 8−10

*Gracilisuchus* (MCZ 4117): 15

*Gualosuchus reigi* (PULR 05): 14

*Gualosuchus reigi* (PVL 4576): 14

*Guchengosuchus* (IVPP V8808-1): 14

*Herrerasaurus* (PVSJ 407): 17−18

*Heterodontosaurus* (AM unnumbered): 12

*Heterodontosuarus* (AM 4765): 13

*Jaxtasuchus salomoni* (SMNS 91083): 16

*Jesairosaurus lehmani* (ZAR 06): ca. 20−21

*Kalisuchus* (QM F8998): ≥14

*Lewisuchus* (PULR 01): 20

*Macrocnemus bessanii* (PIMUZ T4355): 26

*Marasuchus lilloensis* (PVL 3870): ca. 12

*Nicrosaurus kapffi* (SMNS 5727: Hungerbühler, 2000: table 1): 19−21

*Ornithosuchus* (NHMUK PV R2409): 9

*Parasuchus angustifrons* (BSPG 1931 X 502): 16−17

*Pamelaria dolichotrachelos* (ISIR 316/1, based on maxillary and dentary tooth counts): ca. 12−18

*Parasuchus hislopi* (ISIR 42): 21−22

*Petrolacosaurus* (Reisz, 1981: 12): 35

*Planocephalosaurus* (Fraser, 1982: 714): 17

*Prestosuchus chiniquensis* (UFRGS-PV-0156-T): 11

*Prolacerta* (Modesto & Sues, 2004): 24−25

*Prolacertoides jimusarensis* (IVPP V3233): ca. 19

*Proterochampsa barrionuevoi* (MACN-Pv 18165): 12

*Proterochampsa nodosa* (MCP 1694-Pv): 10

*Proterosuchus alexanderi* (NMQR 1484): 27

“*Proterosuchus fergusi*” (SAM-PK-591): >20

*Proterosuchus fergusi* (RC 846): 31

*Proterosuchus goweri* (NMQR 880): 29

*Protorosaurus* (Gottmann-Quesada & Sander, 2009: 141): 28 ± 1

*Pseudochampsa ischigualastensis* (PVSJ 567): ≥15 (but lower than 30)

*Riojasuchus tenuisceps* (PVL 3827): 8

*Sarmatosuchus* (PIN 2865/68, based on the dentary tooth count): ca. <18

*Shansisuchus* (Young, 1964; Wang et al., 2013): 10−13

*Silesaurus opolensis* (Dzik, 2003: 561): 11

*Smilosuchus gregorii* (UCMP 27200): 21

*Smilosuchus adamanensis* (Camp, 1930: 41): 23

*Smilosuchus lithodendrorum* (Camp, 1930: fig. 48): 21−22

*Tanystropheus longobardicus* (Wild, 1973: table 2): 14

*Tasmaniosaurus* (UTGD 54655): >21

*Trilophosaurus buettneri* (Spielmann et al., 2008: 27): 13

*Tropidosuchus romeri* (PVL 4606): ca. 12−13

*Turfanosuchus dabanensis* (IVPP V3237): ca. ≥13

*Uralosaurus magnus* (PIN 2973/71, based on dentary tooth count): <14

*Vancleavea campi* (Nesbitt et al., 2009: 820): 13

*Youngina* (Gow, 1975: 91): ca. 30

*Youngosuchus sinensis* (IVPP V3239): 11

Character 76:

*Aetosauroides scagliai* (PVL 2059): >1.57

*Bentonyx* (BRSUG 27200): 1.06

*Boreopricea* (Tatarinov, 1978: fig. 1a; Benton & Allen, 1997): <0.94

*Chanaresuchus bonapartei* (MCZ 4039): 1.46

*Chanaresuchus bonapartei* (MCZ 4037): 1.19

*Chanaresuchus bonapartei* (PVL 4586): 1.45

“*Chasmatosaurus*” *yuani* (IVPP V4067): 2.58

*Erythrosuchus* (BP/1/5207): 1.42

*Euparkeria* (SAM-PK-13665): 1.26

*Garjainia prima* (PIN 2394/5): ca. 2.75

*Gephyrosaurus* (Evans, 1980: fig. 1): 0.77

GHG 7433MI: >1.81

*Gracilisuchus* (MCZ 4117): ca. 1.35

*Gualosuchus reigi* (PULR 05): 1.84

*Gualosuchus reigi* (PVL 4576): 2.04

*Heterodontosaurus* (Norman et al., 2011: fig. 12): 1.36

*Mesosuchus* (SAM-PK-6536): 0.79

*Nicrosaurus kapffi* (SMNS 5276: Hungerbühler, 1998: fig. 2.15): 2.26

*Ornithosuchus* (Sereno, 1991: fig. 11a): 1.36

*Parasuchus hislopi* (ISIR 42): 1.79

*Petrolacosaurus* (Reisz, 1981: fig. 3): 0.68

*Planocephalosaurus* (Fraser, 1982: fig. 1c): 0.68

*Prestosuchus chiniquensis* (UFRGS-PV-0156-T): 3.08

*Prolacerta* (SAM-PK-K10797): 1.14

*Proterochampsa barrionuevoi* (Dilkes & Arcucci, 2012: fig. 4b): >2.05

*Proterochampsa nodosa* (MCP 1694-Pv): >2.27

*Proterosuchus alexanderi* (NMQR 1484): >1.44

*Proterosuchus fergusi* (BP/1/3993): 3.09

*Proterosuchus fergusi* (GHG 231): 2.66

*Proterosuchus fergusi* (RC 846): 2.78

*Protorosaurus speneri* (USNM 442453, cast of cast of NMK S 180): 1.84

*Pseudochampsa ischigualastensis* (PVSJ 567): ca. 2.46

*Riojasuchus tenuisceps* (PVL 3827): 2.07

*Rhynchosaurus articeps* (NHMUK PV R1236): 1.00

*Shansisuchus shansisuchus* (Wang et al., 2013: fig. 3b): 2.38

*Simoedosaurus lemoinei* (Russell-Sigogneau & Russell, 1978: fig. 1): 1.26

*Smilosuchus lithodendrorum* (Camp, 1930: fig. 2a): 1.05

*Tanystropheus longobardicus* (Wild, 1973: fig. 9a): 1.85

*Turfanosuchus dabanensis* (IVPP V3237): slightly >1.45

*Vancleavea campi* (USNM 508579 cast of GR 138): 0.79

*Youngina* (GHG K 106): 0.92

*Youngosuchus sinensis* (IVPP V3239): 2.96

Discretization by the cluster analysis: (1) 0.68−0.79; (2) 0.92−1.46; (3) 1.78−1.91; (4) 2.04−2.07; (5) 2.26−2.78; (6) 2.96−3.09. (5%=0.12). The character was discretized in three states: (1) 0.68−0.79; (2) 0.92−2.07; (3) 2.26−3.09.

Character 100:

*Acerosodontosaurus* (MNHN 1908-32-57): 1.27

*Amotosaurus* (SMNS 90601): <1.50

*Azendohsaurus madagaskarensis* (Flynn et al., 2010: fig. 13a): 1.59

*Batrachotomus kupferzellensis* (SMNS 52970): >2.58

*Boreopricea* (Tatarinov, 1978: fig. 1b): 0.49

*Cerritosaurus binsfeldi* (CA s/n): 2.87

*Chanaresuchus bonapartei* (PVL 4586): 2.84

*Chanaresuchus bonapartei* (MCZ 4039): 2.68

“*Chasmatosaurus*” *yuani* (IVPP V4067): 5.37

*Cteniogenys* sp. (NHMUK PV R11735): 1.76

*Cuyosuchus* (MCNAM 2669): >4.29

*Erythrosuchus* (BP/1/5207): 3.32

*Euparkeria* (SAM-PK-6047a): 3.17

*Fugusuchus* (Cheng, 1980: fig. 22): 3.19

*Garjainia madiba* (BP/1/5760): 3.76

*Garjainia prima* (PIN 2394/5): 3.77

*Gephyrosaurus* (Evans, 1980: fig. 14a): 1.81

*Gracilisuchus* (PULR 08): 3.64

*Gracilisuchus* (MCZ 4117): 2.23

*Gualosuchus reigi* (PULR 05): 2.90

*Gualosuchus reigi* (PVL 4576): 1.83

*Herrerasaurus* (PVSJ 407): 2.17

*Heterodontosaurus* (AM unnumbered): 0.85

*Heterodontosaurus* (SAM-PK-K337): 0.94

*Jesairosaurus lehmani* (ZAR 08): ca. 2.60

*Lewisuchus* (PULR 01): 3.64

*Mesosuchus* (SAM-PK-6536): 2.95

*Nicrosaurus kapffi* (NHMUK PV 42743): 4.91

*Ornithosuchus longidens* (NHMUK PV R3562): >3.42

*Parasuchus angustifrons* (BSPG 1931 X 502): 4.75

*Parasuchus hislopi* (ISIR 43): 4.24

*Petrolacosaurus* (Reisz, 1981: fig. 2): 0.62

*Planocephalosaurus* (Fraser, 1982: figs. 2a, 3b): 3.45−3.48

*Prestosuchus chiniquensis* (UFRGS-PV-0156-T): 3.07

*Prolacerta* (BP/1/5375): 2.64

*Prolacerta* (SAM-PK-K10797): 2.07

*Prolacerta* (Modesto & Sues, 2004: fig. 8a, BP/1/3575): 3.11

*Proterochampsa barrionuevoi* (Dilkes & Arcucci, 2012: fig. 5d): 1.91

“*Proterosuchus fergusi*” (SAM-PK-591): >1.35

*Proterosuchus alexanderi* (NMQR 1484): 4.07

*Proterosuchus goweri* (NMQR 880): ca. 4.32

*Proterosuchus fergusi* (RC 846): 4.51

*Protorosaurus* (USNM 442453, cast of cast of NMK S 180): 0.70

*Pseudochampsa ischigualastensis* (PVSJ 567): 2.85

*Rhynchosaurus articeps* (NHMUK PV R1236): 2.15

*Riojasuchus tenuisceps* (PVL 3827): 2.27

*Eohyosaurus wolvaardti* (SAM-PK-K10159): 2.46

*Sarmatosuchus* (PIN 2865/68): >2.96

*Shansisuchus shansisuchus* (Young, 1964: fig. 12c): 2.48

*Silesaurus opolensis* (ZPAL AbIII/1930): >1.70

*Simoedosaurus lemoinei* (Russell-Sigogneau & Russell, 1978: fig. 10): 3.25

*Smilosuchus gregorii* (Camp, 1930: fig. 11b): 4.15

*Smilosuchus adamanensis* (Camp, 1930: fig. 11a): 3.45

*Smilosuchus lithodendrorum* (Camp, 1930: fig. 11c): 4.40

*Tanystropheus longobardicus* (PIMUZ T2189): 1.78

*Tropidosuchus romeri* (PVL 4606): 4.1

*Turfanosuchus dabanensis* (IVPP V3237): ca. 3.55

*Vancleavea campi* (USNM 508579 cast of GR 138): 2.37

*Youngina* (Gow, 1975: fig. 5): 2.43

*Youngosuchus sinensis* (IVPP V3239): 2.69

Discretization by the cluster analysis: (1) 0.49−1.27; (2) 1.59−3.77; (3) 4.07−5.37. (5%=0.24).

Character 177:

*Azendohsaurus madagaskarensis* (UA 7-20-99-653): 108º

*Batrachotomus kupferzellensis* (SMNS 52970): 133º

*Boreopricea* (Tatarinov, 1978: fig. 1b): 118º

*Cerritosaurus binsfeldi* (CA s/n): 150º

*Chanaresuchus bonapartei* (MCZ 4039): 132º

*Chanaresuchus bonapartei* (PVL 4586): 136º

*Chanaresuchus bonapartei* (PVL 4575): 134º

“*Chasmatosaurus*” *yuani* (IVPP V4067): 155º

*Cteniogenys* sp. (NHMUK PV R11753): 150º

*Doswellia kaltenbachi* (USNM 214823): 117º

*Erythrosuchus* (BP/1/5207): 128º

*Euparkeria* (SAM-PK-6047a): 129º

*Garjainia prima* (PIN 2394/5): 136º

*Gephyrosaurus* (Evans, 1980: fig. 17d): 91º

*Gracilisuchus* (MCZ 4117): 158º

*Gualosuchus* (PVL 4576): 130º

*Howesia* (SAM-PK-5885): 108º

*Herrerasaurus* (PVSJ 407): 148º

*Heterodontosaurus* (SAM-PK-K337): 112º

*Jesairosaurus lehmani* (ZAR 06): 130º

*Lewisuchus* (PULR 01): 125º

*Marasuchus lilloensis* (Bonaparte, 1975: fig. 3): 110º

*Mesosuchus* (SAM-PK-6536): 116º

*Nicrosaurus kapffi* (SMNS 4379: Hungerbühler, 1998: fig. 2.10): 133º

*Ornithosuchus* (NHMUK PV R2409): ca. 140º

*Parasuchus angustifrons* (BSPG 1931 X 502): 123º

*Paliguana* (AM 3585): 110º

*Pamelaria dolichotrachelos* (ISIR 316/1): 135º

*Parasuchus hislopi* (ISIR 43): 110º

*Planocephalosaurus* (Fraser, 1982: fig. 1a): 129º

*Prestosuchus chiniquensis* (UFRGS-PV-0156-T): 96º

*Prolacerta* (BP/1/471): 95º

*Prolacerta* (BP/1/2675): 126º

*Prolacerta* (BP/1/4504a): 107º

*Prolacerta* (BP/1/5375): 92º

*Prolacerta* (GHG 431): 114º

*Prolacerta* (SAM-PK-K10018): 136º

*Prolacerta* (SAM-PK-K10797): 106º

*Proterosuchus alexanderi* (NMQR 1484): 149º

*Proterosuchus fergusi* (BP/1/4016): 124º

*Proterosuchus fergusi* (SAM-PK-K140): 122º

*Proterosuchus fergusi* (SAM-PK-11208): 125º

*Proterosuchus fergusi* (RC 846): 126º

*Proterosuchus fergusi* (BSPG 1934 VIII 514): 120º

*Proterosuchus fergusi* (GHG 231): 126º

*Proterosuchus goweri* (NMQR 880): 149º

*Protorosaurus* (USNM 442453, cast of cast of NMK S 180): 137º

*Riojasuchus tenuisceps* (PVL 3827): 132º

*Eohyosaurus wolvaardti* (SAM-PK-K10159): 128º

*Sarmatosuchus* (PIN 2865/68): 121º

*Shansisuchus shansisuchus* (Young, 1964: fig. 13a): 130º

*Silesaurus opolensis* (ZPAL AbIII/1930): 135º

*Smilosuchus gregorii* (UCMP 27200): 123º

*Smilosuchus adamanensis* (Camp, 1930: fig. 11a): 135º

*Smilosuchus lithodendrorum* (Camp, 1930: fig. 11c): 137º

*Tanystropheus longobardicus* (Nosotti, 2007: fig. 43, PIMUZ T2484): 95º

*Trilophosaurus buettneri* (Spielmann et al., 2008: fig. 18a): 108º

*Tropidosuchus romeri* (PVL 4601): 126º

*Tropidosuchus romeri* (PVL 4606): 143º

*Turfanosuchus dabanensis* (IVPP V3237): 108º

*Vancleavea campi* (USNM 508579, cast of GR 138): 44º

*Youngina* (SAM-PK-K6205): 131º

*Youngosuchus* (IVPP V3239): 134º

Discretization by the cluster analysis: (1) 41−47º; (2) 91−97º; (3) 106−137º; (4) 143−158º. (5%=5.7).

Character 263:

*Batrachotomus kupferzellensis* (Gower, 1999: fig. 18a): 0.36

*Cerritosaurus binsfeldi* (CA s/n): >0.39

*Chanaresuchus bonapartei* (PVL 4586): 0.51

*Euparkeria* (BP/1/5867): ca. 0.32

*Erythrosuchus* (BP/1/5207): 0.48

*Garjainia prima* (PIN 2394/5): ca. 0.20

*Gracilisuchus* (MCZ 4118): 0.21

*Gualosuchus* (PVL 4576): 0.71

*Herrerasaurus* (PVSJ 407): 0.53

*Heterodontosaurus* (SAM-PK-K1332): 0.16

*Nicrosaurus kapffi* (NHMUK PV 42744: Hungerbühler, 1998: fig. 2.26b): 0.51

*Parasuchus hislopi* (ISIR 42): 0.33

*Prestosuchus chiniquensis* (UFRGS-PV-0152-T): 0.28

*Proterochampsa nodosa* (MCP 1694-Pv): 0.29

*Proterosuchus alexanderi* (NMQR 1484): 0.11

*Proterosuchus fergusi* (RC 59): 0.17

*Proterosuchus fergusi* (RC 846): 0.07

*Proterosuchus fergusi* (SAM-PK-11208): 0.13

*Riojasuchus tenuisceps* (PVL 3827): 0.88

*Silesaurus opolensis* (ZPAL AbIII/1930): 0.24

*Smilosuchus gregorii* (UCMP 27200): 0.44

*Tropidosuchus romeri* (PVL 4601): 0.47

*Turfanosuchus dabanensis* (IVPP V3237): ca. 0.20

*Youngosuchus* (IVPP V3239): 0.18

Discretization by the cluster analysis: (1) 0.07−0.36; (2) 0.44−0.53; (3) 0.71−0.88. (5%=0.04).

Character 266:

*Aetosauroides* (PVL 2059): <0.09

*Azendohsaurus* (FMNH PR 2751): 0.24

*Batrachotomus* (SMNS 80260): ca. 0.18

*Chanaresuchus bonapartei* (PVL 4575): 0.09

*Chanaresuchus bonapartei* (PVL 4586): 0.08

“*Chasmatosaurus ultimus*” (IVPP V2301): <0.14

“*Chasmatosaurus*” *yuani* (IVPP V90002): 0.16

*Dimorphodon* (NHMUK PV R41212-13): 0.11

*Doswellia kaltenbachi* (USNM 186989): 0.09

*Euparkeria* (SAM-PK-K8309): 0.16

*Garjainia madiba* (BP/1/7153): 0.26

*Garjainia prima* (PIN 2394/5-8, 5-9): ca. 0.22

*Garjainia prima* (PIN 951/30): 0.24

*Gephyrosaurus* (Evans, 1980: fig. 41a): 0.11

*Gualosuchus* (PVL 4576): 0.12

*Herrerasaurus* (MACN-Pv 18060): 0.19

*Herrerasaurus* (PVSJ 53): 0.24

*Heterodontosaurus* (AM unnumbered): 0.22

*Heterodontosaurus* (SAM-PK-K1332): 0.23

*Nicrosaurus kapffi* (NHMUK PV R42744): 0.05

*Nundasuchus* (Nesbitt et al., 2014: fig. 3a): <0.19

*Pamelaria dolichotrachelos* (ISIR 316/1): 0.18

*Parasuchus hislopi* (ISIR 42): 0.05

*Petrolacosaurus* (Reisz, 1981: fig. 12): 0.10

*Planocephalosaurus* (Fraser, 1982: plate 70, fig. 2): 0.23

*Prestosuchus chiniquensis* (UFRGS-PV-0152-T): 0.22

*Prolacerta* (BP/1/471): 0.08

*Proterosuchus alexanderi* (NMQR 1484): 0.12

*Proterosuchus fergusi* (RC 846): 0.17

*Proterosuchus fergusi* (BSPG 1934 VIII 514): 0.13

*Rhadinosuchus* (BSPG AS XXV 50): 0.08

*Riojasuchus* (PVL 3827): 0.36

*Sarmatosuchus* (PIN 2865/68-11): 0.22

*Shansisuchus shansisuchus* (Young, 1964: fig. 17c): 0.22

*Silesaurus* (ZPAL AbIII/437/1): 0.16

*Silesaurus* (ZPAL AbIII/1930): 0.18

*Simoedosaurus lemoinei* (MNHN.F.R4014): 0.07

*Smilosuchus gregorii* (UCMP 27200): 0.08

*Tanystropheus* (Nosotti, 2007: fig. 49): 0.11

*Trilophosaurus* (Spielmann et al., 2008: fig. 28b): 0.29

*Turfanosuchus dabanensis* (IVPP V3237): 0.09

*Uralosaurus* (PIN 2973/1): ca. 0.27

*Vancleavea campi* (USNM 508579, cast of GR 138): 0.35

*Youngosuchus* (Young, 1973b: fig. 1, 2): 0.23

Discretization by the cluster analysis: (1) 0.05−0.14; (2) 0.16−0.19; (3) 0.22−0.29; (4) 0.34−0.36. (5%=0.015).

Character 331:

*Aetosauroides scagliai* (PVL 2059): 1.35

*Aetosauroides scagliai* (PVL 2091): 0.91

*Amotosaurus* (SMNS 50830): 6.09−6.80

*Azendohsaurus madagaskarensis* (UA-7-20-99-653): 2.67−2.92

*Batrachotomus kupferzellensis* (SMNS 80288, middle cervical): 0.82

*Batrachotomus kupferzellensis* (MHI 1895, SMNS cast, fifth cervical): 0.97

*Boreopricea* (Benton & Allen, 1997: fig. 6a, PIN 3708/1): 1.92−2.00

*Chanaresuchus bonapartei* (MCZ 4037): 1.03

*Chasmatosuchus rossicus* (PIN 3200/217): 1.62

*Doswellia kaltenbachi* (Weems, 1980: table 3): 2.46

*Eryrhtosuchus* (BP/1/4680, Gower, 2003: table 1): 0.63−0.72

*Eryrhtosuchus* (SAM-PK-K3028, Gower, 2003: table 1): 0.70

*Euparkeria* (SAM-PK-5867): 1.45

*Euparkeria* (UMZC T692): 1.09−1.48

*Gamosaurus* (PIN 3361/13): 1.92

*Garjainia madiba* (BP/1/5360): 0.89

*Garjainia prima* (PIN 2394/5-12, 5-13): 1.07−1.11

*Gephyrosaurus* (Evans, 1980: fig. 4): 1.20

*Gracilisuchus* (PULR 08): 1.76−1.95

*Gracilisuchus* (MCZ 4118): 1.41

*Gualosuchus* (PVL 4576): 1.16−1.24

*Herrerasaurus* (MACN-Pv 18060): 2.23−2.63

*Herrerasaurus* (PVSJ 373): 1.82

*Heterodontosaurus* (SAM-PK-K1332): 1.50−1.53

*Jaxtasuchus salomoni* (SMNS 91083): 2.98

*Jesairosaurus lehmani* (ZAR 07): <2.00

*Lewisuchus* (PULR 01): 2.31

*Macrocnemus bessanii* (PIMUZ T4822): 3.70−4.12

*Marasuchus lilloensis* (PVL 3870): 1.56−1.87

*Marasuchus lilloensis* (PVL 3872): 1.51−2.00

*Mesosuchus* (SAM-PK-5882, fourth cervical): 2.01

*Nicrosaurus kapffi* (SMNS 12671): 0.89

*Nundasuchus* (Nesbitt et al., 2014: fig. 4c): 0.87

*Pamelaria dolichotrachela* (ISI R316): 3.77−3.98

*Pamelaria dolichotrachela* (ISI field number BI/33): 3.80−3.81

*Parasuchus hislopi* (ISI R42: fifith cervical): 0.87

*Petrolacosaurus* (Reisz, 1981: fig. 14): 2.05−2.41

*Prestosuchus chiniquensis* (UFRGS-PV-0152-T): 1.39

*Prolacerta* (BP/1/2675): 3.29−3.50

*Proterochampsa barrionuevoi* (Dilkes & Arcucci, 2012: fig. 11c): 1.56

*Proterosuchus alexanderi* (NMQR 1484): 1.38−1.53

*Proterosuchus fergusi* (BP/1/3993): 1.73

*Proterosuchus fergusi* (SAM-PK-11208): 1.67

*Protorosaurus* (BSPG AS VII 1207; BSPG 1995 I 5, cast of WMsN P 47361): 3.12−3.24

*Rhynchosaurus articeps* (Benton, 1990: fig. 8b): 1.81

*Riojasuchus tenuisceps* (PVL 3827): 1.07−1.16

*Sarmatosuchus* (PIN 2865/68): 0.94−1.08

*Shansisuchus kuyeheensis* (Cheng, 1980: fig. 25): 0.85

*Shansisuchus shansisuchus* (Wang et al., 2013: fig. 4a): 0.77

*Silesaurus opolensis* (ZPAL AbIII/361): 2.18−2.35

*Simoedosaurus lemoinei* (MNHN.F.BL9425): 0.88

*Smilosuchus gregorii* (Camp, 1930: plate III): 0.75−0.77

*Tanystropheus longobardicus* (PIMUZ T2818, fourth cervical): 14.25

*Trilophosaurus buettneri* (Spielmann et al., 2008: appendix 10): 1.84−2.50

*Tropidosuchus romeri* (PVL 4601): 2.07−2.15

*Yarasuchus deccanensis* (ISI R334): 2.05−3.10

*Youngosuchus* (IVPP V3239): 1.30

Discretization by the cluster analysis: (1) 0.63−2.67; (2) 2.92−4.12; (3) 6.09−6.80; (4) 14.16−14.33. (5%=0.17). *Amotosaurus* and *Tanystropheus* were not included in the cluster analysis and the 5% variation calculation.

Character 351:

*Acerosodontosaurus* (MNHN 1908-32-57): 1.71

*Aenigmastropheus* (UMZC T836): 1.35

*Aetosauroides scagliai* (PVL 2052): 0.92−0.93

*Aetosauroides scagliai* (PVL 2059): 1.28−1.34

*Aetosauroides scagliai* (PVL 2073): 1.10

*Amotosaurus* (SMNS 54784b): 1.93

*Archeopelta arborensis* (CPEZ-239a): 0.88

*Azendohsaurus madagaskarensis* (UA 8-26-98-250): 1.38

*Azendohsaurus madagaskarensis* (UA 8-26-98-265): 1.51

*Batrachotomus kupferzellensis* (SMNS 80309): 1.09

*Batrachotomus kupferzellensis* (SMNS 80296): 1.18

*Boreopricea* (PIN 3708/1): 1.56−1.67

*Chalishevia* (PIN 4188/98): 0.95

*Chanaresuchus bonapartei* (MCZ 4037): 1.04−1.19

*Chanaresuchus bonapartei* (PVL 4575): 1.60

“*Chasmatosaurus*” *yuani* (IVPP V2719): 1.18−1.30

*Cteniogenys* sp. (NHMUK PV R11787): 2.00

*Cuyosuchus* (MCNAM 2669): 1.25−1.39

“*Dongusia colorata*” (PIN 268/2): 1.10

*Doswellia kaltenbachi* (Weems, 1980: table 4): 1.93−2.00

*Eorasaurus olsoni* (PIN 156/100): ca. 1.65

*Erythrosuchus* (NHMUK PV R3592 large, Gower, 2003: table 1): 0.55

*Erythrosuchus* (BP/1/4680, Gower, 2003: table 1): 0.48

*Erythrosuchus* (SAM-PK-Kun-no, Gower, 2003: table 1): 0.45−0.60

*Euparkeria* (UMZC T692): 1.39−1.89

*Herrerasaurus* (PVSJ 373): 1.02−1.30

*Jaxtasuchus salomoni* (SMNS 91352): 1.66

*Jesairosaurus lehmani* (ZAR 10): 2.45

*Garjainia madiba* (BP/1/7135): 1.08

*Garjainia prima* (PIN 2394/5-14, 5-16): 0.80−0.96

*Gracilisuchus* (MCZ 4118): 1.48−1.50

*Gualosuchus* (PVL 4576): 0.95

*Lewisuchus* (PULR 01): 1.73−1.85

*Macrocnemus bessanii* (PIMUZ T2472): 1.40−1.53

*Macrocnemus bessanii* (PIMUZ T4355): 1.52−1.62

*Marasuchus lilloensis* (PVL 3870): 1.90−2.19

*Marasuchus lilloensis* (PVL 3872): 1.32−1.71

*Mesosuchus* (SAM-PK-6046: second vertebra of the axial series): 1.26

*Nicrosaurus kapffi* (SMNS 12671): 0.89

*Pamelaria dolichotrachela* (ISI R316): 1.38−1.39

*Pamelaria dolichotrachela* (ISI field number BI/33): 1.64

*Pamelaria dolichotrachela* (ISI field number BI/53): 1.42−1.55

*Parasuchus hislopi* (ISIR 42): 1.20

*Petrolacosaurus* (Reisz, 1981: fig. 15a, b): 1.76

*Prestosuchus chiniquensis* (UFRGS-PV-0152-T): 0.90−0.93

*Prolacerta* (BP/1/2675, first and second dorsals): 1.78

*Proterosuchus alexanderi* (NMQR 1484): 1.00

*Proterosuchus fergusi* (SAM-PK-K140): 0.96−1.01

*Protorosaurus* (BSPG 1995 I 5, cast of WMsN P 47361; ZMR MB R2172): 1.70−1.73

*Pseudochampsa ischigualastensis* (PVSJ 567): 1.40

*Rhynchosaurus articeps* (SHYMS 2): 1.83

*Riojasuchus tenuisceps* (PVL 3827): 0.84−0.92

*Shansisuchus kuyeheensis* (Cheng, 1980: fig. 26): 1.00−1.10

*Shansisuchus shansisuchus* (Wang et al., 2013: fig. 4a): 0.72

*Shansisuchus shansisuchus* (Young, 1964: fig. 23a-c): 0.68−0.84

*Silesaurus opolensis* (ZPAL AbIII/1302): 1.37

*Simoedosaurus lemoinei* (MNHN.F.BR12208): 1.23

*Smilosuchus gregorii* (UCMP 26699): 0.89−0.98

*Tanystropheus longobardicus* (PIMUZ T2817, first to third dorsal): 2.39−2.74

*Tasmaniosaurus* (UTGD 54655, anterior-middle dorsal): 1.29

*Trilophosaurus buettneri* (Spielmann et al., 2008: appendix 10, postaxial vertebrae 8−11): 1.23−1.79

*Tropidosuchus romeri* (PVL 4601, 12−13^th^ presacral): 1.34−1.51

*Vancleavea campi* (Nesbitt et al., 2009: fig. 11b): 1.67

*Yarasuchus deccanensis* (ISI R334): 1.56−1.82

*Youngina* (BP/1/3859): 1.77−1.85

Discretization by the cluster analysis: (1) 0.45−1.10; (2) 1.18−2.00; (3) 2.19−2.74. (5%=0.11). In this case, the difference between ranges of the states (0) and (1) is lower than the variation of 5%, but it was discretized to capture the information provided by the anteroposteriorly short vertebrae of some erythrosuchids and suchians.

Character 352:

*Acerosodontosaurus* (MNHN 1908-32-57): 1.50

*Aetosauroides scagliai* (PVL 2073, dorsals 14−16): 1.38−1.54

*Amotosaurus* (SMNS 54783): 1.60−1.95

*Archeopelta arborensis* (CPEZ-239a): 1.31

*Azendohsaurus madagaskarensis* (UA 8-29-98-325): 0.97

*Azendohsaurus madagaskarensis* (UA 7-20-99-654): 1.02

*Azendohsaurus madagaskarensis* (UA 8-27-98-270): 0.77

*Batrachotomus kupferzellensis* (SMNS 80300): 1.14

*Chanaresuchus bonapartei* (MCZ 4037): 1.23−1.35

“*Chasmatosaurus*” *yuani* (IVPP V2719): 1.28−1.31

*Chasmatosuchus rossicus* (PIN 3200/212): 1.16

*Cteniogenys* sp. (NHMUK PV R11788): 1.76

*Cuyosuchus* (MCNAM 2669): 1.18−1.21

*Dimorphodon* (NHMUK PV R41212-13): 1.38−1.48

*Doswellia kaltenbachi* (Weems, 1980: table 4): 1.86−2.00

*Erythrosuchus* (NHMUK PV R3592 large, Gower, 2003: table 1): 0.83

*Erythrosuchus* (NHMUK PV R3592 small, Gower, 2003: table 1): 0.78

*Erythrosuchus* (SAM-PK-905, Gower, 2003: table 1): 0.79

*Euparkeria* (SAM-PK-6047A): 1.26−1.48

*Garjainia madiba* (BP/1/6232aj): 1.00

*Garjainia prima* (Huene, 1960: plate 13, figs. 13, 14): 1.00−1.23

*Gephyrosaurus* (Evans, 1980: fig. 7): 2.39

*Gracilisuchus* (MCZ 4118): 1.30−1.39

*Gualosuchus* (PVL 4576): 1.39−1.49

*Herrerasaurus* (MACN-Pv 18060): 0.85

*Herrerasaurus* (MCZ 7064): 0.66

*Herrerasaurus* (PVSJ 373): 0.85

*Heterodontosaurus* (AM unnumbered): 1.49−1.70

*Howesia* (SAM-PK-5886): 1.25−1.36

*Jesairosaurus lehmani* (ZAR 12): 1.84−2.04

*Jesairosaurus lehmani* (ZAR 14): 1.27

*Lagerpeton chanarensis* (PVL 4625): 1.54−2.42

*Lewisuchus* (PULR 01): 2.03

*Macrocnemus bessanii* (PIMUZ T4822): 2.46

*Marasuchus lilloensis* (PVL 3870): 1.59−1.77

*Mesosuchus* (SAM-PK-6046): 1.18−1.38

*Noteosuchus* (AM 3591, ninth vertebra of preserved series): 1.39

*Nundasuchus* (Nesbitt et al., 2014: fig. 4o): 0.83−0.91

*Ornithosuchus* (Walker, 1964: fig. 8j): 1.67

*Pamelaria dolichotrachela* (ISI field number BI/33): 1.63

*Parasuchus hislopi* (ISI R42): 1.19−1.29

*Petrolacosaurus* (Reisz, 1981: fig. 15e): 1.32−1.33

*Planocephalosaurus* (Fraser & Walkden, 1984: fig. 7b): 1.96

*Prestosuchus chiniquensis* (UFRGS-PV-0152-T): 0.87−0.93

*Prolacerta* (BP/1/2675, 20^th^ presacral): 1.70

*Proterochampsa barrionuevoi* (Trotteyn, 2011a: fig. 5): 1.60

*Protorosaurus* (BSPG 1995 I 5, cast of WMsN P 47361): 1.38

*Pseudochampsa ischigualastensis* (PVSJ 567): 1.73

*Riojasuchus tenuisceps* (PVL 3827): 1.00−1.01

*Sarmatosuchus* (PIN 2865/68-23, 68-24): 0.87−0.94

*Shansisuchus shansisuchus* (Young, 1964: fig. 23a-c): 1.07−1.26

*Silesaurus opolensis* (ZPAL AbIII/1832/1): 1.13

*Simoedosaurus lemoinei* (Sigogneau-Russell, 1981: fig. 11c−e): 1.07−1.18

*Smilosuchus gregorii* (UCMP 26699): 0.85−0.94

*Trilophosaurus buettneri* (Spielmann et al., 2008: appendix 10, presacral vertebrae 21−24): 1.36−1.79

*Tropidosuchus romeri* (PVL 4601, 21th presacral): 1.72

*Yarasuchus deccanensis* (ISI R334): 1.20

*Youngina* (BP/1/3859): 1.73

Discretization by the cluster analysis: (1) 0.66−1.39; (2) 1.48−1.86; (3) 1.95−2.04; (4) 2.39−2.46. (5%=0.09).

Character 377:

*Aetosauroides scagliai* (PVL 2052): >1.12

*Amotosaurus* (SMNS 90600): ca. 1.62

*Azendohsaurus madagaskarensis* (UA 8-25-98-220): 0.67

*Azendohsaurus madagaskarensis* (UA 8-29-97-169): 0.62

*Chanaresuchus bonapartei* (PVL 4575): 1.68

“*Chasmatosaurus*” *yuani* (Young, 1936: fig. 6): 2.20

*Cuyosuchus* (MCNAM 2669): 0.85−1.08

*Doswellia kaltenbachi* (USNM 244214): 2.72

*Gracilisuchus* (PVL 4597): 0.72−0.81

*Herrerasaurus* (PVL 2566: first caudal): 1.04

*Jesairosaurus lehmani* (ZAR 09): 0.79

*Macrocnemus bessanii* (PIMUZ T4355): ca. 1.64

*Marasuchus lilloensis* (PVL 3871): ca. 0.65

*Mesosuchus* (SAM-PK-7416: first caudal): ca. 1.51

*Noteosuchus* (AM 3591, first and second caudal): 1.53−1.58

*Petrolacosaurus* (Reisz, 1981: fig. 15h, i): 2.46

*Prolacerta* (Gow, 1975: fig. 22, 1^st^-2^nd^ caudals): 0.80−1.01

*Proterosuchus alexanderi* (NMQR 1484): 0.71−0.89

*Protorosaurus* (BSPG 1995 I 5, cast of WMsN P 47361): 0.35

*Pseudochampsa ischigualastensis* (PVSJ 567): 1.18

*Silesaurus opolensis* (Dzik, 2003: fig. 12a): 0.92

*Simoedosaurus lemoinei* (MNHN.F.R2241): 1.20

*Tanystropheus longobardicus* (Wild, 1973: fig. 59d): >0.92

*Trilophosaurus buettneri* (Spielmann et al., 2008: fig. 50): 0.74

*Tropidosuchus romeri* (PVL 4601, third caudal): ca. 1.45

*Youngina* (BP/1/3859): 1.15

Discretization by the cluster analysis: (1) 0.29−0.41; (2) 0.62−1.20; (3) 1.51−1.68; (4) 2.20−2.72. (5%=0.12).

Character 379:

*Aetosauroides scagliai* (PVL 2073): >1.00

*Azendohsaurus madagaskarensis* (UA 8-29-97-169): 1.36

*Batrachotomus kupferzellensis* (SMNS 80337): 3.42

*Chanaresuchus bonapartei* (PVL 4575): 1.49

“*Chasmatosaurus*” *yuani* (IVPP V4067): 2.09

*Cuyosuchus* (MCNAM 2669): 1.94−2.46

*Dorosuchus* (PIN 1579/64): >1.15

*Erythrosuchus* (NHMUK PV R3592, Gower, 2003: table 1): >1.25

*Euparkeria* (SAM-PK-K8050): 2.21−2.36

*Doswellia kaltenbachi* (Weems, 1980: table 5): 0.47−0.53

*Garjainia madiba* (BP/1/5360): >1.30

*Garjainia prima* (Huene, 1960: plate 13, fig. 17): 1.94

*Gracilisuchus* (PVL 4597): 1.68

*Heterodontosaurus* (SAM-PK-K1332): 2.03

*Howesia* (SAM-PK-5886): 2.65−2.98

*Jesairosaurus lehmani* (ZAR 09): 1.61−1.74

*Lagerpeton chanarensis* (PVL 4625): 1.47

*Lewisuchus* (PULR 01): 0.84

*Mesosuchus* (SAM-PK-6046: second to fourth caudal): 2.99−3.54

*Nicrosaurus kapffi* (SMNS 12671): 2.98

*Noteosuchus* (AM 3591): 2.11

*Ornithosuchus* (Walker, 1964: fig. 8j, 2^nd^-4^th^ caudal): 1.81−2.05

*Ornithosuchus* (Walker, 1964: fig. 8l, 1^st^-2^nd^ caudal): 1.45−1.81

*Pamelaria dolichotracela* (ISI R316/12): 1.45

*Petrolacosaurus* (Reisz, 1981: fig. 15h): 1.22

*Prestosuchus chiniquensis* (UFRGS-PV-0152-T): 1.61

*Prolacerta* (Gow, 1975: fig. 22, 1^st^-2^nd^ caudals): 1.51−1.54

*Proterosuchus alexanderi* (NMQR 1484): 1.52−1.96

*Protorosaurus* (BSPG 1995 I 5, cast of WMsN P 47361): 2.13

*Rhynchosaurus articeps* (BRLSI M20a: caudals 1−5): 1.52−2.56

*Silesaurus opolensis* (ZPAL Ab/unnumbered): 1.79

*Simoedosaurus lemoinei* (MNHN.F.R2241): 0.66

*Smilosuchus gregorii* (UCMP 26699): 2.44−2.53

*Tanystropheus longobardicus* (Wild, 1973: fig. 59a): 1.48

*Trilophosaurus buettneri* (Spielmann et al., 2008: fig. 50e, h): 1.60−2.14

*Tropidosuchus romeri* (PVL 4601, first and second caudals): 0.96−1.07

*Turfanosuchus dabanensis* (IVPP V3237): 1.91

*Yarasuchus deccanensis* (ISI R334): 3.54

*Youngina* (Currie, 1981: fig. 1d): 1.19

Discretization by the cluster analysis: (1) 0.66−2.21; (2) 2.36−2.65; (3) 2.92−3.05; (4) 3.42−3.54. (5%=0.144).

Character 387:

*Aetosauroides scagliai* (PVL 2073): 5.02

*Amotosaurus* (SMNS 50830): 1.46

*Amotosaurus* (SMNS 54810): 1.33

*Azendohsaurus madagaskarensis* (UA 9-8-98-501): 4.88

*Batrachotomus kupferzellensis* (SMNS 80271): 6.36

*Boreopricea funerea* (PIN 3708/1): ca. 2.28

*Chanaresuchus bonapartei* (PVL 4575): 7.92

*Chanaresuchus bonapartei* (MCZ 4035): 8.97

“*Chasmatosaurus*” *yuani* (IVPP V2719): ca. 2.04

*Dimorphodon* (NHMUK PV R41212-13): ca. 8.80

*Erythrosuchus* (SAM-PK-905): 5.60

*Erythrosuchus* (NHMUK PV R3592): 5.90

*Euparkeria* (SAM-PK-5867): 4.44

*Garjainia madiba* (BP/1/7152): 4.45

*Garjainia prima* (PIN 2394/5-32, 5-33): 3.73−3.74

*Garjainia prima* (Huene, 1960: table 14, fig. 10): 4.17

*Gracilisuchus* (PULR 08): >5.62

*Gualosuchus* (PVL 4576): >6.46

*Guchengosuchus* (Peng, 1991: fig. 7): 5.28

*Herrerasaurus* (PVSJ 053): 8.64

*Heterodontosaurus* (SAM-PK-K1332): 9.79

*Jesairosaurus lehmani* (ZAR 09): > 7.00

*Lewisuchus* (PULR 01): ca. 9.10

*Macrocnemus bessanii* (PIMUZ T4355): 1.47

*Mesosuchus* (SAM-PK-6536): >3.56

*Nundasuchus* (Nesbitt et al., 2014: table 1): 5.32

*Ornithosuchus* (Walker, 1964: fig. 9f): 8.76

*Pamelaria dolichotrachela* (ISI R316): <2.50

*Parasuchus hislopi* (Chatterjee, 1978: fig. 10a): 4.34

*Planocephalosaurus* (Fraser & Walkden, 1984: fig. 13a): ca. 5.10

*Prestosuchus chiniquensis* (UFRGS-PV-0629-T): 4.87

*Prolacerta* (BP/1/2675): ca. 1.79

*Proterosuchus alexanderi* (NMQR 1484): 2.13

*Protorosaurus* (BSPG 1995 I 5, cast of WMsN P 47361): 2.61

*Rhynchosaurus articeps* (SHYMS 2): 4.69

*Sarmatosuchus* (PIN 2865/68): 2.10

*Shansisuchus kuyeheensis* (Cheng, 1980: fig. 27): 5.47

*Shansisuchus shansisuchus* (Young, 1964: fig. 26a): 6.08

*Shansisuchus shansisuchus* (Young, 1964: fig. 26b): 6.00

*Shansisuchus shansisuchus* (Young, 1964: fig. 26b): 5.81

*Shansisuchus shansisuchus* (Young, 1964: fig. 26d): 5.96

*Silesaurus opolensis* (ZPAL AbIII/2534): 11.31

*Simoedosaurus lemoinei* (MNHN.F.BR1009): 5.00

*Simoedosaurus lemoinei* (MNHN.F.BR1013): 6.27

*Smilosuchus gregorii* (UCMP 26699): 6.29

*Tanystropheus longobardicus* (Nosotti, 2007: fig 20): 1.40

*Tanystropheus longobardicus* (Wild, 1973: plate 17): 1.23

*Trilophosaurus buettneri* (Spielmann et al., 2008: fig. 65b): >6.15

*Tropidosuchus romeri* (PVL 4601): 6.73

*Vancleavea campi* (Nesbitt et al., 2009: fig. 12): 4.46

*Youngina* (BP/1/3859): 3.25

*Youngosuchus* (IVPP V3239): >5.40

Discretization by the cluster analysis: (1) 1.23−6.73; (2) 7.92−10.45. (5%=0.46).

Character 410:

*Aetosauroides scagliai* (PVL 2073): 0.11

*Azendohsaurus madagaskarensis* (UA 8-27-98-271): 0.08

“*Chasmatosaurus*” *yuani* (IVPP V4067): 0.22

*Doswellia kaltenbachi* (USNM 244214): 0.14

*Euparkeria* (SAM-PK-5867): <0.09

*Garjainia prima* (PIN 2394/5-34): 0.21

*Jesairosaurus lehmani* (ZAR 09): 0.10

*Macrocnemus bessanii* (PIMUZ T4355): 0.09

*Mesosuchus* (SAM-PK-6536): 0.10

*Nicrosaurus kapffi* (SMNS 5705/1): 0.27

*Parasuchus hislopi* (ISI collection): 0.22

*Petrolacosaurus* (Reisz, 1981: fig. 17b): 0.11

*Prestosuchus chiniquensis* (UFRGS-PV-0152-T): 0.20

*Prolacerta* (BP/1/2675): 0.08

*Proterosuchus alexanderi* (NMQR 1484): 0.25

*Proterosuchus fergusi* (GHG 363): 0.24

*Protorosaurus* (Gottmann-Quesada & Sander, 2009: fig. 19): <0.17

*Rhynchosaurus articeps* (NHMUK PV R1239): 0.11

*Simoedosaurus lemoinei* (MNHN.F.R1413): 0.12

*Tasmaniosaurus* (UTGD 54655): 0.07

*Trilophosaurus buettneri* (Gregory, 1945: fig. 8b): 0.13

*Youngina* (SAM-PK-K7710): 0.13

Discretization by the cluster analysis: (1) 0.07−0.14; (2) 0.20−0.27 (5%=0.01).

Character 416:

*Aetosauroides scagliai* (PVL 2073): 0.34

*Aetosauroides scagliai* (PVL 2091): 0.58

*Amotosaurus* (SMNS 54783): 0.22

*Azendohsaurus madagaskarensis* (UA 7-13-99-578): 0.53

*Azendohsaurus madagaskarensis* (UA 8-29-97-151): 0.53

*Batrachotomus kupferzellensis* (SMNS 80276): 0.41

*Chanaresuchus bonapartei* (PVL 4575): 0.38

“*Chasmatosaurus*” *yuani* (IVPP V2719): 0.48

“*Chasmatosaurus*” *yuani* (IVPP V4067): 0.50

*Cuyosuchus* (MCNAM 2669): 0.41

*Dimorphodon* (Padian, 1983: table 1, YPM 350): 0.37

*Erythrosuchus* (SAM-PK-905): 0.70

*Euparkeria* (SAM-PK-5867): 0.37

*Euparkeria* (SAM-PK-7696): 0.46

*Euparkeria* (SAM-PK-8050): 0.38

*Euparkeria* (SAM-PK-13666): 0.35

*Garjainia madiba* (BP/1/5360): 0.63

*Garjainia prima* (PIN specimen): 0.57

*Gracilisuchus* (CRILAR #079-2011): 0.23

*Gualosuchus* (PVL 4576): 0.35

*Herrerasaurus* (MACN-PV 18060): 0.29

*Heterodontosaurus* (SAM-PK-K1332): 0.25

*Jaxtasuchus salomoni* (SMNS 91002): 0.31

*Jesairosaurus lehmani* (ZAR 09): 0.39

*Koilamasuchus* (MACN-Pv 18119): >0.41

*Lewisuchus* (PULR 01): ca. 0.28

*Macrocnemus bessanii* (PIMUZ T2472): 0.21

*Marasuchus lilloensis* (PVL 3871): >0.18

*Nicrosaurus kapffi* (SMNS unnumbered): 0.38

*Nundasuchus* (Nesbitt et al., 2014: table 1): 0.54

*Pamelaria dolichotrachela* (ISI R316/51): 0.44

*Pamelaria dolichotrachela* (ISI R324/35, 36): 0.44−0.45

*Parasuchus hislopi* (ISI collection): 0.38

*Petrolacosaurus* (Reiz, 1981: 43): 0.27

*Prestosuchus chiniquensis* (UFRGS-PV-0152-T): 0.38

*Prolacerta* (BP/1/2675): 0.32

*Proterochampsa barrionuevoi* (Trotteyn, 2011a: table 2): 0.41

*Protorosaurus* (Gottmann-Quesada & Sander, 2009: appendix I, FG 2666/2004b): 0.20

*Protorosaurus* (Gottmann-Quesada & Sander, 2009: appendix I, WMsN P 47361): 0.33

*Rhynchosaurus articeps* (NHMUK PV R1239): 0.38

*Riojasuchus tenuisceps* (PVL 3826): 0.46

*Shansisuchus kuyeheensis* (Cheng, 1980: fig. 28): 0.57

*Shansisuchus shansisuchus* (Young, 1964: table 7, 27a): 0.60

*Shansisuchus shansisuchus* (Young, 1964: table 7, 27b): 0.60

*Shansisuchus shansisuchus* (Young, 1964: table 7, 27c): 0.58

*Shansisuchus shansisuchus* (Young, 1964: table 7, 27d): 0.62

*Silesaurus opolensis* (ZPAL AbIII/361): 0.22

*Silesaurus opolensis* (ZPAL AbIII/362): 0.21

*Silesaurus opolensis* (ZPAL AbIII/452): 0.20

*Simoedosaurus lemoinei* (MNHN.F.BR1236): 0.34

*Simoedosaurus lemoinei* (MNHN.F.CRL2203): 0.34

*Smilosuchus gregorii* (USNM 18313): 0.34

*Tanystropheus longobardicus* (PIMUZ T2817): 0.20

*Tanystropheus longobardicus* (Nosotti, 2007: fig. 7): 0.20

*Trilophosaurus buettneri* (Spielmann et al., 2008: fig. 66a): 0.31

*Tropidosuchus romeri* (PVL 4601): 0.24

*Turfanosuchus dabanensis* (IVPP V3237): 0.39

*Vancleavea campi* (Parker & Barton, 2008: fig. 8.4): 0.28

*Yarasuchus deccanensis* (ISI R334/53): 0.40

*Youngina* (BP/1/3859): 0.32

*Youngosuchus* (IVPP V3239): 0.41

Discretization by the cluster analysis: (1) 0.20−0.41; (2) 0.44−0.70 (5%=0.025).

Character 424:

*Aetosauroides scagliai* (PVL 2073): 0.33

*Aetosauroides scagliai* (PVL 2091): 0.30

*Azendohsaurus madagaskarensis* (UA 8-29-97-151): 0.30

*Batrachotomus kupferzellensis* (SMNS 80276): 0.46

*Chanaresuchus bonapartei* (PVL 4575): 0.31

“*Chasmatosaurus*” *yuani* (IVPP V2719): 0.37

“*Chasmatosaurus*” *yuani* (IVPP V4067): 0.42

*Cuyosuchus* (Rusconi, 1951: fig. 38c): 0.48

*Dimorphodon* (Padian, 1983: fig. 8, YPM 350): 0.46

*Erythrosuchus* (SAM-PK-905): 0.55

*Euparkeria* (SAM-PK-5867): 0.38

*Garjainia madiba* (BP/1/5360): 0.43

*Garjainia prima* (PIN specimen): 0.48

*Gracilisuchus* (CRILAR #079-2011): 0.49

*Herrerasaurus* (MACN-PV 18060): 0.52

*Heterodontosaurus* (SAM-PK-K1332): 0.42

*Jaxtasuchus salomoni* (SMNS 91002): 0.36

*Lewisuchus* (PULR 01): >0.37

*Marasuchus lilloensis* (PVL 3871): ca. 0.40

*Nicrosaurus kapffi* (SMNS unnumbered): 0.32

*Nundasuchus* (Nesbitt et al., 2014: fig. 7b): 0.43

*Pamelaria dolichotrachela* (ISI R316/51): 0.42

*Pamelaria dolichotrachela* (ISI R324/35, 36): 0.45−0.46

*Parasuchus hislopi* (ISI collection): 0.35

*Petrolacosaurus* (Reisz, 1981: fig. 19): 0.17

*Prestosuchus chiniquensis* (UFRGS-PV-0152-T): 0.34

*Prolacerta* (BP/1/2675): 0.27

*Proterochampsa barrionuevoi* (Trotteyn, 2011a: fig. 9): 0.25

*Protorosaurus* (BSPG 1995 I 5, cast of WMsN P 47361): 0.26

*Rhynchosaurus articeps* (NHMUK PV R1239): 0.34

*Riojasuchus tenuisceps* (PVL 3826): 0.43

*Silesaurus opolensis* (ZPAL AbIII/452): 0.39

*Shansisuchus kuyeheensis* (Cheng, 1980: fig. 28): 0.46

*Shansisuchus shansisuchus* (Young, 1964: table 7, 27a): 0.40

*Shansisuchus shansisuchus* (Young, 1964: table 7, 27b): 0.40

*Shansisuchus shansisuchus* (Young, 1964: table 7, 27c): 0.42

*Shansisuchus shansisuchus* (Young, 1964: table 7, 27d): 0.48

*Simoedosaurus lemoinei* (MNHN.F.BL9626): 0.38

*Smilosuchus gregorii* (USNM 18313): 0.28

*Trilophosauurs buettneri* (Spielmann et al., 2008: fig. 66d): 0.24

*Turfanosuchus dabanensis* (IVPP V3237): 0.37

*Vancleavea campi* (Parker & Barton, 2008: fig. 8.4): 0.33

*Yarasuchus deccanensis* (ISI R334/53): 0.49

*Youngina* (SAM-PK-K7710): 0.28

*Youngosuchus* (IVPP V3239): 0.43

Discretization by the cluster analysis: (1) 0.16−0.18; (2) 0.24−0.49; (3) 0.52−0.55 (5%=0.02).

Character 435:

*Aetosauroides scagliai* (PVL 2059): 0.90

*Aetosauroides scagliai* (PVL 2073): 0.73

*Boreopricea* (Benton & Allen, 1997: 941, 942): 0.79

*Chanaresuchus bonapartei* (Romer, 1972b: 12): 0.92

“*Chasmatosaurus*” *yuani* (Young, 1978: table 1; IVPP V4067): 0.76

*Erythrosuchus* (SAM-PK-905): 0.75

*Euparkeria* (SAM-PK-5867): 0.84

*Euparkeria* (SAM-PK-13666): 0.83

*Herrerasaurus* (PVSJ 407): 0.92

*Heterodontosaurus* (SAM-PK-K1332): 0.70

*Macrocnemus bessanii* (Rieppel, 1989a: table 2, PIMUZ T2477): 0.95

*Macrocnemus bessanii* (Rieppel, 1989a: table 2, PIMUZ T2472): 0.92

*Macrocnemus bessanii* (Rieppel, 1989a: table 2, PIMUZ T4355): 0.96

*Marasuchus lilloensis* (PVL 3871): >0.72

*Ornithosuchus* (Huene, 1914: 17): 0.79

*Pamelaria dolichotrachela* (ISI R316/51, 53): 0.89

*Parasuchus hislopi* (Chatterjee, 1978: table 2): 0.67

*Petrolacosaurus* (Reisz, 1981: 45): 0.92

*Prolacerta* (Gow, 1975: 111, BP/1/2675): 0.88

*Proterochampsa barrionuevoi* (Trotteyn, 2011a: table 2): 0.76

*Protorosaurus* (Gottmann-Quesada & Sander, 2009: appendix I, FG 2666/2004b): 0.84

*Protorosaurus* (Gottmann-Quesada & Sander, 2009: appendix I, NHMW 1943I4): 0.79

*Protorosaurus* (Gottmann-Quesada & Sander, 2009: appendix I, WMsN P 47361): 0.86

*Rhynchosaurus articeps* (NHMUK PV R1238): 0.71

*Rhynchosaurus articeps* (SHYMS 6): 0.84

*Riojasuchus tenuisceps* (Bonaparte, 1972): ca. 0.75

*Silesaurus opolensis* (ZPAL AbIII/361): 1.12

*Tanystropheus longobardicus* (Nosotti, 2007: table 6, MSNM BES SC 1018): 0.66

*Tanystropheus longobardicus* (Nosotti, 2007: table 4, MSNM BES SC 265): 0.70

*Trilophosaurus buettneri* (Spielmann et al., 2008: fig. 72d, appendix 10, TMM 31025-140): 0.81

*Tropidosuchus romeri* (Arcucci, 1990: 373): 0.78

*Vancleavea campi* (Nesbitt et al., 2009: fig. 13): 0.62

*Youngina* (Gow, 1975: 97): 0.78

*Youngina* (SAM-PK-K7710): 0.70

*Youngosuchus* (IVPP V3239): 0.97

Discretization by the cluster analysis: (1) 0.62−0.66; (2) 0.69−0.92; (3) 0.95−0.97; (4) 1.12−1.17. (5%=0.03).

Character 446:

*Amotosaurus* (SMNS 54783): 0.34

*Boreopricea* (Tatarinov, 1978: fig. 2): 0.74

GHG 7433MI: 0.39

*Herrerasaurus* (PVSJ 373): 0.38

*Heterodontosaurus* (SAM-PK-K1332): 0.34

*Jaxtasuchus salomoni* (SMNS 91352): 0.58

*Macrocnemus bessanii* (PIMUZ T2472): 0.43

*Noteosuchus* (AM 3591): 0.45

*Pamelaria dolichotrachela* (ISI R136): 0.56

*Proterosuchus fergusi* (SAM-PK-K140): 0.59

*Protorosaurus* (Gottmann-Quesada & Sander, 2009: appendix I, Nat. Kab. 191): 0.97

*Tanystropheus longobardicus* (Nosotti, 2007: tables 5, 6, MSNM BES SC 1018): 0.38

*Trilophosaurus buettneri* (Spielmann et al., 2008: figs.74a, 91a): 0.59

*Vancleavea campi* (Nesbitt et al., 2009: 831, 836): 0.75

*Youngina* (based on extrapolations between the forelimb of BP/1/3859 and the hindlimb of SAM-PK-K7710d): 0.54

Discretization by the cluster analysis: (1) 0.34−0.39; (2) 0.43−0.45; (3) 0.54−0.59; (4) 0.74−0.75; (5) 0.95−0.98. (5%=0.03). The last three clusters were merged together because the last two were restricted to a single terminal.

Character 448:

*Acerosodontosaurus* (MNHN 1908-32-57): 0.44

*Amotosaurus* (SMNS 54783): 0.26

*Azendohsaurus madagaskarensis* (UA 7-16-99-607): 0.53

*Dimorphodon* (NHMUK PV R41212-13): <0.12

*Erythrosuchus* (NHMUK PV R3592): 0.74

*Euparkeria* (SAM-PK-13666): 0.52−0.60

*Herrerasaurus* (PVSJ 373): 0.40

*Herrerasaurus* (PVSJ 380): 0.50

*Heterodontosaurus* (SAM-PK-K1332): 0.31

*Jaxtasuchus salomoni* (SMNS 91352): 0.64

*Mesosuchus browni* (SAM-PK-6046): 0.48

*Noteosuchus* (AM 3591): 0.48

*Ornithosuchus* (Walker, 1964: fig. 10f): 0.33

*Pamelaria dolichotrachela* (ISI R136/54): 0.31

*Petrolacosaurus* (Reisz, 1981): 0.48

*Prolacerta* (BP/1/2675): 0.36

*Proterosuchus fergusi* (SAM-PK-K140): 0.50

*Protorosaurus* (BSPG 1995 I 5, cast of WMsN P 47361): 0.50

*Rhynchosaurus articeps* (SHYMS 4): 0.43

*Rhynchosaurus articeps* (SHYMS 6): 0.39

*Riojasuchus tenuisceps* (PVL 3827): 0.37

*Shansisuchus shansisuchus* (Young, 1964: table 9): 0.58−0.63

*Tanystropheus longobardicus* (Nosotti, 2007: fig. 23, MSNM BES SC 1018): 0.38

*Trilophosaurus buettneri* (Spielmann et al., 2008: fig. 74a): 0.50

*Vancleavea campi* (Nesbitt et al., 2009: fig. 13): 0.45

*Youngina* (BP/1/3859): 0.42

Discretization by the cluster analysis: (1) 0.26−0.33; (2) 0.36−0.45; (3) 0.48−0.53; (4) 0.58−0.64; (5) 0.73−0.75. (5%=0.02).

Character 458:

*Acerosodontosaurus* (MNHN 1908-32-57): 0.33

*Aetosauroides scagliai* (PVL 2073): 0.39

*Archeopelta arborensis* (CPEZ-239a): 0.43

*Chanaresuchus bonapartei* (Romer, 1972b: plate 1): 0.38

*Cuyosuchus* (MCNAM 2669): <0.38

*Dimorphodon* (NHMUK PV R1034): 0.12

*Dorosuchus* (PIN 1579/61): 0.47

*Lagerpeton chanarensis* (PVL 4619): 0.26

*Gracilisuchus* (PVL 4597): ca. 0.24−0.30

*Herrerasaurus* (PVL 2566): 0.37

*Heterodontosaurus* (AM unnumbered): 0.25

*Heterodontosaurus* (SAM-PK-K1332): 0.30

*Macrocnemus bessanii* (PIMUZ T2472): 0.24

*Macrocnemus bessanii* (Besano II specimen): 0.25

*Marasuchus lilloensis* (PVL 3870): 0.31

*Mesosuchus* (Haughton, 1922: 86, 87; SAM-PK-6046): 0.56

*Noteosuchus* (Carroll, 1976: figs. 2, 3): 0.38

*Ornithosuchus* (NHMUK PV R3561): 0.34

*Pamelaria dolichotrachela* (ISI R136/49, 50, 55): 0.21

*Parasuchus hislopi* (Chatterjee, 1978: figs. 12, 13): 0.24

*Prestosuchus chiniquensis* (UFRGS-PV-0629-T): 0.47

*Prolacerta* (BP/1/2676): 0.41

*Riojasuchus tenuisceps* (PVL 3828): 0.35

*Silesaurus opolensis* (ZPAL AbIII/361): >0.28

*Tanystropheus longobardicus* (Nosotti, 2007: fig. 27, MSNM BES SC 1018): 0.29

*Trilophosaurus buettneri* (Spielmann et al., 2008: fig. 76a, appendix 10: TMM 31025-140): >0.31

*Turfanosuchus dabanensis* (IVPP V3237): ca. 0.40

*Yarasuchus deccanensis* (ISI R334/56, 67): 0.17

*Youngina* (BP/1/3859): 0.40

Discretization by the cluster analysis: (1) 0.12−0.17; (2) 0.21−0.47; (3) 0.54−0.57 (5%=0.022).

Character 463:

*Acerosodontosaurus* (MNHN 1908-32-57): 1.24

*Aetosauroides scagliai* (PVL 2059): 0.31

*Aetosauroides scagliai* (PVL 2073): 0.35

*Amotosaurus* (SMNS 90544): 1.08

*Azendohsaurus madagaskarensis* (UA 8-30-98-375): 1.04

*Azendohsaurus madagaskarensis* (UA 9-5-98-448): 1.03

*Azendohsaurus madagaskarensis* (UA 8-29-97-155): 0.98

*Batrachotomus kupferzellensis* (SMNS 80273): 1.13

*Chanaresuchus bonapartei* (MCZ 4035): 0.85

“*Chasmatosaurus*” *yuani* (Young, 1936: fig. 10): ca. 0.92

*Cuyosuchus* (Rusconi, 1951: fig. 31a): 0.91

*Dimorphodon* (NHMUK PV R1034): 1.18

*Dorosuchus* (PIN 1579/61): 1.03

*Doswellia kaltenbachi* (USNM 244214): 1.22

*Erythrosuchus* (NHMUK PV R3592): 0.87

*Erythrosuchus* (SAM-PK-905): 0.90

*Euparkeria* (SAM-PK-7696): 1.21

*Garjainia madiba* (BP/1/5525): 1.19

*Garjainia prima* (PIN specimen): 0.98

*Gracilisuchus* (PVL 4597): 1.11

*Herrerasaurus* (PVL 2566): 0.79

*Herrerasaurus* (MCZ 4381): 0.94

*Herrerasaurus* (MLP-61-VIII-2-2): 0.94

*Heterodontosaurus* (SAM-PK-K1332): 1.19

*Howesia* (NHMUK PV R5872, cast of SAM-PK-5886): ca. 0.92

*Koilamasuchus* (MACN-Pv 18119): 0.55−0.85

*Lagerpeton chanarensis* (PVL 4619): 0.89

*Macrocnemus bessanii* (Besano II specimen): 1.00

*Marasuchus lilloensis* (PVL 3870): 0.89

*Mesosuchus* (SAM-PK-7416): 0.82

*Noteosuchus* (AM 3591): 1.11

*Ornithosuchus* (Walker, 1964: fig. 11f): 1.00

*Pamelaria dolichotrachela* (ISI R136/49, 50): 1.00

*Parasuchus hislopi* (ISI collection): 1.02

*Petrolacosaurus* (Reisz, 1981: fig. 18): 1.00

*Prestosuchus chiniquensis* (UFRGS-PV-0152-T): 0.96

*Prestosuchus chiniquensis* (UFRGS-PV-0629-T): 0.96

*Prolacerta* (BP/1/2676): >1.07

*Planocephalosaurus* (Fraser & Walkden, 1984: fig. 17b): 0.92

*Riojasuchus tenuisceps* (PVL 3827): 1.36

*Riojasuchus tenuisceps* (PVL 3828): 1.52

*Shansisuchus shansisuchus* (Young, 1964: fig. 30b): 1.34

*Silesaurus opolensis* (ZPAL AbIII/361): 1.02

*Silesaurus opolensis* (ZPAL AbIII/404/1):

*Simoedosaurus lemoinei* (MNHN.F.BR12090): 1.24

*Smilosuchus gregorii* (UCMP 26699): 1.03

*Tanystropheus longobardicus* (Nosotti, 2007: fig. 24, MSNM BES SC 1018): 1.32

*Trilophosaurus buettneri* (Spielmann et al., 2008: fig. 76a): 0.82

*Turfanosuchus dabanensis* (IVPP V3237): 1.34

*Vancleavea campi* (Nesbitt et al., 2009: fig. 14b): 0.63

*Yarasuchus deccanensis* (ISI R334/56): 1.11

*Yarasuchus deccanensis* (ISI R334): 1.04

*Youngina* (BP/1/3859): 0.45

Discretization by the cluster analysis: (1) 0.31−0.63; (2) 0.79−1.24; (3) 1.31−1.37; (4) 1.49−1.55. (5%=0.06).

Character 472:

*Acerosodontosaurus* (Currie, 1980: fig. 7): 1.82

*Aetosauroides scagliai* (PVL 2052): 2.19

*Aetosauroides scagliai* (PVL 2073): 2.14

*Amotosaurus* (SMNS 50830): ca. 2.17

*Chanaresuchus bonapartei* (PVL 4575): 1.86

*Cuyosuchus* (Rusconi, 1951: fig. 37): 2.47

*Dimorphodon* (NHMUK PV R41212-13): 1.90

*Doswellia kaltenbachi* (USNM 244214): 1.75

*Erythrosuchus* (SAM-PK-905): 1.52

*Euparkeria* (SAM-PK-7696): 2.27

*Garjainia prima* (PIN specimen): 1.97

*Gracilisuchus* (PVL 4597): 2.37

*Herrerasaurus* (MCZ 4381): 3.43

*Herrerasaurus* (PVL 2566): 4.22

*Heterodontosaurus* (SAM-PK-K1332): 4.87

*Howesia* (SAM-PK-5886): 1.65

*Lagerpeton chanarensis* (PVL 4619): 1.58

*Macrocnemus* *bessanii* (Besano II specimen): 1.44

*Marasuchus lilloensis* (PVL 3870): 2.84

*Mesosuchus* (SAM-PK-7416): 1.66

*Mesosuchus* (SAM-PK-6046): ca. 1.35

*Noteosuchus* (Carroll, 1976: figs. 1, 3h): 1.69

*Ornithosuchus* (Walker, 1964: fig. 11f): 3.94

*Pamelaria dolichotrachela* (ISI R136/49, 50): 1.64

*Parasuchus hislopi* (Chatterjee, 1978: fig. 12a): 1.15

*Petrolacosaurus* (Reisz, 1981: fig. 18): 2.16

*Planocephalosaurus* (Fraser & Walkden, 1984: fig. 16): 2.58

*Prestosuchus chiniquensis* (UFRGS-PV-0629-T): 2.36

*Prolacerta* (BP/1/2676): 2.01

*Riojasuchus tenuisceps* (PVL 3827): 3.40

*Silesaurus opolensis* (ZPAL AbIII/361): 4.59

*Smilosuchus gregorii* (Long & Murry, 1995: fig. 31a): 1.61

*Tanystropheus longobardicus* (Nosotti, 2007: fig. 24, MSNM BES SC 1018): 2.34

*Trilophosaurus buettneri* (Spielmann et al., 2008: figs. 76a, 78a): 1.39

*Tropidosuchus romeri* (PVL 4601): 1.92

*Turfanosuchus dabanensis* (IVPP V3237): 3.06

*Yarasuchus deccanensis* (ISI R334): 2.84

*Youngina* (BP/1/3859): 1.20

Discretization by the cluster analysis: (1) 1.15−2.58; (2) 2.84−3.43; (3) 3.94−4.87. (5%=0.186).

Character 478:

*Aetosauroides scagliai* (PVL 2052): 0.86

*Aetosauroides scagliai* (PVL 2073): 0.77

*Azendohsaurus madagaskarensis* (UA 8-30-98-375): 0.97

*Batrachotomus kupferzellensis* (SMNS 80269): 0.34

*Chanaresuchus bonapartei* (PVL 4575): 1.80

*Cuyosuchus* (MCNAM 2669): >0.85

*Doswellia kaltenbachi* (USNM 244214): 1.69

*Erythrosuchus* (NHMUK PV R3592): 0.88

*Erythrosuchus* (SAM-PK-905): 0.96

*Garjainia prima* (PIN specimen): 1.12

*Gracilisuchus* (PVL 4597): 0.27

*Herrerasaurus* (PVL 2566): 0.41

*Lagerpeton chanarensis* (PVL 4619): 1.68

*Marasuchus lilloensis* (PVL 3870): 0.56

*Mesosuchus* (SAM-PK-6046): 1.94

*Noteosuchus* (Carroll, 1976: fig. 1): 1.54

*Nundasuchus* (Nesbitt et al., 2014: fig. 8b): slightly >1.07

*Ornithosuchus* (Walker, 1964: fig. 11h): 0.47

*Pamelaria dolichotrachela* (ISI R136/49, 50): 1.19

*Prestosuchus chiniquensis* (UFRGS-PV-0629-T): 0.43

*Riojasuchus tenuisceps* (PVL 3827): 0.59

*Shansisuchus shansisuchus* (Young, 1964: fig. 29e): 1.13

*Silesaurus opolensis* (Dzik, 2003: fig. 12d): 0.49

*Smilosuchus gregorii* (UCMP 26699): 1.28

*Tropidosuchus romeri* (PVL 4601): 1.48

*Turfanosuchus dabanensis* (IVPP V3237): ca. 0.52

*Yarasuchus deccanensis* (ISI R334): 0.56

Discretization by the cluster analysis: (1) 0.27−0.59; (2) 0.77−0.97; (3) 1.12−1.28; (4) 1.48−1.94. (5%=0.08).

Character 482:

*Aetosauroides scagliai* (PVL 2052): 1.79

*Aetosauroides scagliai* (PVL 2073): 1.77

*Amotosaurus* (SMNS 50830): ca. 2.17

*Chanaresuchus bonapartei* (MCZ 4035): 1.65

*Cuyosuchus* (MCNAM 2669): 2.19

*Dimorphodon macronyx* (NHMUK PV R41212-13): 1.72

*Doswellia kaltenbachi* (USNM 244214): 1.66

*Erythrosuchus* (SAM-PK-905): 1.90

*Euparkeria* (SAM-PK-7696): 2.72

*Garjainia madiba* (BP/1/5525): 2.40

*Garjainia prima* (PIN specimen): 2.43

*Gephyrosaurus* (Evans, 1981: fig. 20): ca. 2.00

*Gracilisuchus* (PVL 4597): 1.99−2.36

*Herrerasaurus* (MCZ 4381): 1.64

*Heterodontosaurus* (SAM-PK-K1332): 4.4

*Howesia* (NHMUK PV R5872, cast of SAM-PK-5886): ca. 1.79

*Lagerpeton chanarensis* (PVL 4619): 2.17

*Macrocnemus* *bessanii* (Besano II specimen): 1.55

*Marasuchus lilloensis* (PVL 3870): 2.18

*Mesosuchus* (SAM-PK-6046): 1.24

*Noteosuchus* (Carroll, 1976: figs. 1, 3h): 2.31

*Ornithosuchus* (Walker, 1964: fig. 11g): 2.96

*Pamelaria dolichotrachela* (ISI R136/49, 50): 1.56

*Parasuchus hislopi* (Chatterjee, 1978: fig. 12a): 1.55

*Petrolacosaurus* (Reisz, 1981: fig. 18): 2.72

*Planocephalosaurus* (Fraser & Walkden, 1984: fig. 16): 2.35

*Prestosuchus chiniquensis* (UFRGS-PV-0629-T): 2.24

*Prolacerta* (BP/1/2676): 1.92

*Rhynchosaurus articeps* (SHYMS 5): 1.58

*Silesaurus opolensis* (ZPAL AbIII/361): 3.53

*Smilosuchus gregorii* (Long & Murry, 1995: fig. 31a): 2.03

*Tanystropheus longobardicus* (Nosotti, 2007: fig. 25, MSNM BES SC 1018): 2.50

*Trilophosaurus buettneri* (Spielmann et al., 2008: fig. 82a): 1.66

*Tropidosuchus romeri* (PVL 4601): 1.71

*Vancleavea campi* (Nesbitt et al., 2009: fig. 14b, c): 2.43

*Yarasuchus deccanensis* (ISI R334): 2.78

*Youngina* (BP/1/3859): 1.04

Discretization by the cluster analysis: (1) 1.04−1.24; (2) 1.55−2.50; (3) 2.72−3.53; (4) 4.31−4.48. (5%=0.17).

Character 489:

*Aetosauroides scagliai* (PVL 2073): 1.62

*Boreopricea* (Tatarinov, 1978: 511; Benton & Allen, 1997: 941, 942): 1.19−1.25

*Chanaresuchus bonapartei* (PVL 4575): 1.63

*Chanaresuchus bonapartei* (MCZ 4035): 1.51

“*Chasmatosaurus*” *yuani* (IVPP V2719): 1.50

“*Chasmatosaurus*” *yuani* (table 1, IVPP V4067): 1.38

*Cuyosuchus* (MCNAM 2669): >1.31

*Dimorphodon* (Padian, 1983: table 1, YPM 9182): 0.94

*Euparkeria* (SAM-PK-5867): 1.47

*Euparkeria* (SAM-PK-7696): 1.31

*Gualosuchus* (PVL 4576): 1.46

*Herrerasaurus* (MACN-Pv 18060): 1.86−1.96

*Heterodontosaurus* (SAM-PK-K1332): 1.35

*Jaxtasuchus salomoni* (SMNS 91002): 1.62

*Macrocnemus bessanii* (Rieppel, 1989a: table 2, PIMUZ T2477): 1.32

*Macrocnemus bessanii* (Rieppel, 1989a: table 2, PIMUZ T2472): 1.25

*Macrocnemus bessanii* (Rieppel, 1989a: table 2, PIMUZ T4355): 1.23

*Marasuchus lilloensis* (PVL 3871): 1.44

*Nundasuchus* (Nesbitt et al., 2014: table 1): 1.37

*Pamelaria dolichotrachela* (ISI R136/51, 55): 1.09

*Parasuchus hislopi* (Chatterjee, 1978: table 2): 1.32

*Petrolacosaurus* (Peabody, 1952: table 4, estimated femoral length of 1427 based on the ratio between the radii of 1427 and 1428): 1.15

*Prestosuchus chiniquensis* (UFRGS-PV-0152-T): 1.53

*Proterochampsa barrionuevoi* (Trotteyn, 2011a: table 2): 1.67

*Protorosaurus* (Gottmann-Quesada & Sander, 2009: appendix I, BSPG AS VII 1207): 1.55

*Protorosaurus* (Gottmann-Quesada & Sander, 2009: appendix I, NHMW 1943I4): 1.41

*Silesaurus opolensis* (ZPAL AbIII/362): 1.39

*Tanystropheus longobardicus* (Nosotti, 2007: tables 4, 5, MSNM BES SC 265): 1.44

*Tanystropheus longobardicus* (Wild, 1973: plate 7, specimen g): 1.53

*Tanystropheus longobardicus* (Wild, 1973: plate 7, specimen a): 1.42

*Tanystropheus longobardicus* (PMIUZ T2817): 1.45

*Trilophosauurs buettneri* (Spielmann et al., 2008: appendix 10, TMM 31025-140): 1.21

*Tropidosuchus romeri* (PVL 4601): 1.40

*Turfanosuchus dabanensis* (IVPP V3237): 1.56

*Vancleavea campi* (Nesbitt et al., 2009: figs. 13, 15): 1.74

*Yarasuchus deccanensis* (ISI R334/53, 67): 1.64

*Youngina* (BP/1/3859): 1.48

Discretization by the cluster analysis: (1) 0.92−0.97; (2) 1.09−1.56; (3) 1.62−1.74; (4) 1.86−1.96 (5%=0.05).

Character 490:

*Acerosodontosaurus* (MNHN 1908-32-57): 1.14

*Aetosauroides scagliai* (PVL 2052): 1.67

*Aetosauroides scagliai* (PVL 2073): 1.73

*Boreopricea* (Benton & Allen, 1997: fig. 2): 1.46

*Chanaresuchus bonapartei* (PVL 4575): 2.00

“*Chasmatosaurus yuani*” (IVPP V2719): 1.24

“*Chasmatosaurus yuani*” (IVPP V4067): 1.08−1.18

*Cuyosuchus* (MCNAM 2669): 1.24

*Euparkeria* (SAM-PK-5867): 1.53

*Euparkeria* (SAM-PK-7696): 1.65

*Gracilisuchus* (CRILAR #079-2011): 0.95

*Gualosuchus* (PULR 05): 1.57

*Heterodontosaurus* (SAM-PK-K1332): 1.29

*Jaxtasuchus salomoni* (SMNS 91002): 1.93

*Macrocnemus bessanii* (PIMUZ T2472): 1.80

*Macrocnemus bessanii* (PIMUZ T4355): 1.29

*Marasuchus lilloensis* (PVL 3871): 1.57

*Nundasuchus* (Nesbitt et al., 2014: table 1): 1.32

*Pamelaria dolichotrachela* (ISI R136/51, 55): 1.01

*Parasuchus hislopi* (Chatterjee, 1978: table 2): 1.18

*Prestosuchus chiniquensis* (UFRGS-PV-0152-T): 1.59

*Proterochampsa barrionuevoi* (Trotteyn, 2011a: fig. 3): ca. 1.09

*Rhynchosaurus articeps* (NHMUK PV R1239): 1.50

*Riojasuchus tenuisceps* (PVL 3828): 1.70

*Tanystropheus longobardicus* (Nosotti, 2007: plate IV, MSNM BES SC 1018): 1.18

*Trilophosauurs buettneri* (Spielmann et al., 2008: appendix 10, TMM 31025-140): 1.13

*Turfanosuchus dabanensis* (IVPP V3237): 1.46

*Vancleavea campi* (Nesbitt et al., 2009: figs. 13, 15): 1.21

*Yarasuchus deccanensis* (ISI R334/53, 67): 1.26

*Youngina* (SAM-PK-K7710): 1.09

Discretization by the cluster analysis: (1) 0.95−1.01; (2) 1.08−1.32; (3) 1.46−1.80; (4) 1.93−2.00. (5%=0.05).

Character 510:

*Aetosauroides scagliai* (PVL 2073): 0.26−0.27

*Archeopelta arborensis* (CPEZ-239a): 0.26

*Azendohsaurus madagaskarensis* (UA 9-8-98-502): 0.29

*Batrachotomus kupferzellensis* (SMNS 52970): 0.24

*Boreopricea* (Benton & Allen, 1997: 942): 0.22

*Chanaresuchus bonapartei* (PVL 4575): 0.16

*Chanaresuchus bonapartei* (MCZ 4035): 0.20

“*Chasmatosaurus*” *yuani* (IVPP V2719): 0.31

“*Chasmatosaurus*” *yuani* (IVPP V4067): 0.32

*Cuyosuchus* (MCNAM 2669): <0.29

*Dimorphodon* (Padian, 1983: table 1, YPM 9182): 0.14

*Dongusuchus* (PIN 952/15-1): 0.18

*Dongusuchus* (PIN 952/15-2): 0.18

*Dorosuchus* (PIN 1579/61): 0.27

*Doswellia kaltenbachi* (USNM 186989): ca. 0.18

*Erythrosuchus* (NHMUK PV R3592): 0.40

*Euparkeria* (SAM-PK-5867): 0.19

*Euparkeria* (SAM-PK-5867): 0.24

*Euparkeria* (SAM-PK-6047B): 0.27

*Euparkeria* (SAM-PK-7868): 0.20

*Garjainia madiba* (BP/1/5767): 0.32

*Garjainia prima* (PIN specimen): 0.34

*Gracilisuchus* (PVL 4597): 0.08

*Gualosuchus* (PULR 05): ca. 0.24

*Gualosuchus* (PVL 4576): ca. 0.22

*Herrerasaurus* (MACN-Pv 18060): 0.17

*Herrerasaurus* (PVL 2566): 0.22

*Herrerasaurus* (PVSJ 373): 0.21

*Lagerpeton chanarensis* (MCZ 4121): 0.20

*Lagerpeton chanarensis* (PVL 4619): 0.21

*Marasuchus lilloensis* (PVL 3870): 0.15

*Mesosuchus* (SAM-PK-7416): 0.31

*Noteosuchus* (Carroll, 1976: fig. 5b): 0.29

*Nundasuchus* (Nesbitt et al., 2014: table 1): 0.30

*Ornithosuchus* (Walker, 1964: fig. 12c): 0.18

*Pamelaria dolichotrachela* (ISI R136/55): 0.29

*Parasuchus hislopi* (Chatterjee, 1978: ISI collection): 0.22

*Petrolacosaurus* (Reisz, 1981: 45): 0.30

*Prestosuchus chiniquensis* (UFRGS-PV-0152-T): 0.23

*Prolacerta* (BP/1/2676): 0.23

*Proterosuchus fergusi* (SAM-PK-K140): 0.36

*Proterochampsa barrionuevoi* (Trotteyn, 2011a: table 2): 0.18

*Protorosaurus* (SMNS 55387, cast of Simon/Bartholomäus specimen): 0.23

*Riojasuchus tenuisceps* (PVL 3827): 0.33

*Riojasuchus tenuisceps* (PVL 3828): 0.24

*Shansisuchus shansisuchus* (Young, 1964: table 8, 31a): 0.30

*Shansisuchus shansisuchus* (Young, 1964: table 8, 31b): 0.28

*Shansisuchus shansisuchus* (Young, 1964: table 8, 31c): 0.32

*Silesaurus opolensis* (ZPAL AbIII/361/25): 0.16

*Simoedosaurus lemoinei* (MNHN.F.R3404): 0.24

*Simoedosaurus lemoinei* (MNHN.F.BR1348): 0.24

*Smilosuchus gregorii* (Long & Murry, 1995: fig. 36b): 0.20

*Tanystropheus longobardicus* (SMNS unnumbered): 0.13

*Trilophosauurs buettneri* (Spielmann et al., 2008: appendix 10, TMM 31025-140): 0.21

*Trilophosauurs buettneri* (Spielmann et al., 2008: appendix 10, TMM 31025-694): 0.21

*Trilophosauurs buettneri* (Spielmann et al., 2008: appendix 10, TMM 31025-067): 0.15

*Trilophosauurs buettneri* (Spielmann et al., 2008: appendix 10, TMM 31025-826): 0.19

*Trilophosauurs buettneri* (Spielmann et al., 2008: appendix 10, TMM 31025-67-RR): 0.18

*Tropidosuchus romeri* (PVL 4601): 0.13

*Turfanosuchus dabanensis* (IVPP V3237): 0.19

*Vancleavea campi* (AMNH 30884, cast): 0.22

*Yarasuchus deccanensis* (ISI R334): 0.21

*Youngina* (BP/1/3859): 0.11

Discretization by the cluster analysis: (1) 0.08−0.11; (2) 0.13−0.24; (3) 0.26−0.36; (4) 0.39−0.41. (5%=0.02).

Character 516:

*Aetosauroides scagliai* (PVL 2052): <0.95

*Aetosauroides scagliai* (PVL 2073): >0.74

*Amotosaurus* (SMNS 54810): 1.03

*Boreopricea* (PIN 3708/1 and Tatarinov, 1978: 511): 0.80

*Chanaresuchus bonapartei* (MCZ 4035): 0.74

*Chanaresuchus bonapartei* (PVL 4575): 0.74

“*Chasmatosaurus*” *yuani* (IVPP V2719): 0.88

“*Chasmatosaurus*” *yuani* (IVPP V4067): 0.63

*Cuyosuchus* (MCNAM 2669): <0.79

*Dimorphodon* (Padian, 1983: table 1, YPM 9182): 1.44

*Dorosuchus* (PIN 1579/61): 0.74

*Euparkeria* (SAM-PK-5867): 0.86

*Euparkeria* (SAM-PK-7696): 0.81

*Euparkeria* (SAM-PK-7707): 0.83

*Gracilisuchus* (PVL 4597): 0.91

*Gualosuchus* (PVL 4576): 0.74

*Herrerasaurus* (MACN-Pv 18060): 0.90

*Herrerasaurus* (PVL 2566): 0.87

*Herrerasaurus* (PVSJ 373): 0.91

*Heterodontosaurus* (SAM-PK-K1332): 1.14

*Jaxtasuchus salomoni* (SMNS 91352): 0.72

*Jesairosaurus lehmani* (ZAR 15): <0.98

*Lagerpeton chanarensis* (PULR 06): 1.19

*Lagerpeton chanarensis* (PVL 4619): 1.17

*Macrocnemus bessanii* (Rieppel, 1989a: table 2, AIII/208): 1.01

*Macrocnemus bessanii* (Rieppel, 1989a: table 2, PIMUZ T2477): 1.06

*Macrocnemus bessanii* (Rieppel, 1989a: table 2, PIMUZ T2472): 1.08

*Macrocnemus bessanii* (Rieppel, 1989a: table 2, PIMUZ T4355): 1.11

*Macrocnemus bessanii* (Peyer, 1937: 91, Alla Cascina): 0.97

*Macrocnemus bessanii* (Peyer, 1937: 91, Besano II): 0.99

*Macrocnemus bessanii* (Peyer, 1937: 94, Tre Fontane 1936): 1.05−1.06

*Marasuchus lilloensis* (PVL 3870): 1.11−1.19

*Marasuchus lilloensis* (PVL 3871): 1.22−1.27

*Mesosuchus* (Haughton, 1922: 87, SAM-PK-6046): 0.98

*Mesosuchus* (Dilkes, 1998: 518, SAM-PK-7416): 0.83

*Noteosuchus* (Carroll, 1976: 48): 0.90

*Nundasuchus* (Nesbitt et al., 2014: table 1, length of fibula instead of tibia): 0.71

*Pamelaria dolichotrachela* (ISI R136/55, 56): 0.84

*Parasuchus hislopi* (Chatterjee, 1978: table 2): 0.64

*Petrolacosaurus* (Peabody, 1952: table 4 for KUVP 1428): 1.16

*Prestosuchus chiniquensis* (UFRGS-PV-0629-T): 0.82

*Prolacerta* (Gow, 1975: 111, BP/1/2676): 1.07

*Proterochampsa barrionuevoi* (Trotteyn, 2011a: table 2): 0.70

*Protorosaurus* (Gottmann-Quesada & Sander, 2009: appendix I, IPB R 535): 0.89

*Protorosaurus* (Gottmann-Quesada & Sander, 2009: appendix I, Nat. Kab. 191): 0.87

*Protorosaurus* (Gottmann-Quesada & Sander, 2009: appendix I, NHMW 1943I4): 0.93

*Protorosaurus* (Gottmann-Quesada & Sander, 2009: appendix I, Simon/Bartholomäus specimen): 0.91

*Pseudochampsa ischigualastensis* (PVSJ 567): 0.83−0.89

*Rhynchosaurus articeps* (SHYMS 5): 0.96

*Riojasuchus tenuisceps* (PVL 3827): 0.83

*Silesaurus opolensis* (ZPAL AbIII/362): 0.91

*Tanystropheus longobardicus* (Nosotti, 2007: table 5, MSNM BES SC 265): 0.85−0.88

*Tanystropheus longobardicus* (PIMUZ T2817): 0.74

*Tasmaniosaurus* (UTGD 54655): <0.89

*Trilophosauurs buettneri* (Spielmann et al., 2008: fig. 85b): 0.82

*Tropidosuchus romeri* (PVL 4601): 0.92

*Vancleavea campi* (Nesbitt et al., 2009: fig. 15): 0.48

*Yarasuchus deccanensis* (ISI R334): 0.71

*Yarasuchus deccanensis* (ISI unnumbered): 0.84

*Youngina* (SAM-PK-K7710a): 1.00

*Youngina* (SAM-PK-K7710b): 0.95

*Youngina* (SAM-PK-K7710d): 0.95

*Youngina* (SAM-PK-K7710e): 1.00

Discretization by the cluster analysis: (1) 0.46−0.51; (2) 0.60−0.65; (3) 0.70−1.27; (4) 1.41−1.46. (5%=0.05).

Character 556:

*Aetosauroides scagliai* (PVL 2052): 1.05

*Aetosauroides scagliai* (PVL 2073): 1.03

*Amotosaurus* (SMNS 54783a): 0.85

*Azendohsaurus madagaskarensis* (UA 8-25-98-231): 0.61

*Erythrosuchus* (NHMUK PV R3592): 0.58

*Euparkeria* (UMZC T692): 0.48

*Gracilisuchus* (PVL 4597): 0.89

*Herrerasaurus* (PVSJ 373): 0.30

*Howesia* (Carroll, 1976: fig. 8): 0.56

*Macrocnemus bessanii* (PIMUZ T4822): 1.20

*Macrocnemus bessanii* (Rieppel, 1989a: fig. 8c, PIMUZ T2473): 1.08

*Marasuchus lilloensis* (Sereno & Arcucci, 1994: fig. 11): 0.42

*Mesosuchus* (SAM-PK-7416): 0.55

*Nundasuchus* (Nesbitt et al., 2014: fig. 12a): 0.63

*Pamelaria dolichotracela* (ISIR collection): 0.48

*Parasuchus hislopi* (Chatterjee, 1978: ISI collection): 0.59

*Petrolacosaurus* (Reisz, 1981: fig. 24): 0.87

*Prestosuchus chiniquensis* (UFRGS-PV-0152-T): 0.64

*Prolacerta* (BP/1/2676): 0.56

*Proterosuchus alexanderi* (NMQR 1484): 0.44

*Protorosaurus* (SMNS 55387, cast of the Simon/Bartholomäus specimen): 0.75

*Riojasuchus tenuisceps* (PVL 3827): 0.73

*Silesaurus opolensis* (ZPAL AbIII/361/18, 20): 0.42−0.43

*Smilosuchus gregorii* (USNM 18313): 0.54

*Tanystropheus longobardicus* (Nosotti, 2007: fig. 63, PIMUZ T2480): 0.58

*Trilophosauurs buettneri* (Spielmann et al., 2008: fig. 91b): 0.71

*Tropidosuchus romeri* (PVL 4601): 0.44

*Vancleavea campi* (Nesbitt et al., 2009: fig. 17): 0.46

*Youngina* (Broom, 1921: fig. 20): 0.74

Discretization by the cluster analysis: (1) 0.28−0.33; (2) 0.42−0.48; (3) 0.54−1.22. (5%=0.045).

Character 567:

*Aetosauroides scagliai* (PVL 2052): 0.46

*Amotosaurus* (SMNS 54783): 0.37

*Chanaresuchus bonapartei* (Romer, 1972b: fig. 2): 0.55

*Dimorphodon* (Padian, 1983: table 1, YPM 350): 0.31

*Euparkeria* (SAM-PK-7696): 0.43

*Gracilisuchus* (PVL 4597): 0.43

*Herrerasaurus* (PVSJ 373): 0.52

*Heterodontosaurus* (SAM-PK-K1332): 0.53

*Jaxtasuchus salomoni* (SMNS 91352): 0.59

*Lagerpeton chanarensis* (PULR 06): 0.53

*Lagerpeton chanarensis* (PVL 4619): 0.53

*Macrocnemus bessanii* (Rieppel, 1989a: table 2, A III/208): 0.52

*Macrocnemus bessanii* (Rieppel, 1989a: table 2, PIMUZ T2473): 0.42

*Macrocnemus bessanii* (Rieppel, 1989a: table 2, PIMUZ T2477): 0.52

*Macrocnemus bessanii* (Rieppel, 1989a: table 2, PIMUZ T2472): 0.47

*Macrocnemus bessanii* (Rieppel, 1989a: table 2, PIMUZ T4355): 0.45

*Macrocnemus bessanii* (Peyer, 1937: 91, Alla Cascina specimen): 0.54

*Macrocnemus bessanii* (Peyer, 1937: 94, Tre Fontane specimen 1936): 0.51

*Marasuchus lilloensis* (PVL 3870): 0.56

*Marasuchus lilloensis* (PVL 3871): 0.58

*Mesosuchus* (SAM-PK-7416): 0.53

*Noteosuchus* (AM 3591): 0.48

*Nundasuchus* (Nesbitt et al., 2014: table 1, fibula used instead of tibia): 0.43

*Pamelaria dolichotracelos* (ISIR 316/56, 58): 0.37

*Parasuchus hislopi* (Chatterjee, 1978: figs. 12, 13): 0.57

*Petrolacosaurus* (Peabody, 1952: fig. 6a): 0.43

*Prestosuchus chiniquensis* (UFRGS-PV-0152-T): 0.41

*Prolacerta* (BP/1/2676): 0.38

*Proterochampsa barrionuevoi* (Trotteyn, 2011a: table 2): >0.50

*Protorosaurus* (Gottmann-Quesada & Sander, 2009: appendix I, IPB R 535): 0.54

*Protorosaurus* (Gottmann-Quesada & Sander, 2009: appendix I, Nat. Kab. 191): 0.21

*Protorosaurus* (Gottmann-Quesada & Sander, 2009: appendix I, NHMW 1943I4): 0.45−0.51

*Protorosaurus* (Gottmann-Quesada & Sander, 2009: appendix I, Simon/Bartholomäus specimen): 0.47

*Pseudochampsa ischigualastensis* (PVSJ 567): 0.39−0.43

*Rhynchosaurus articeps* (SHYMS 5): 0.51

*Rhynchosaurus articeps* (NHMUK PV R1238): 0.52

*Riojasuchus tenuisceps* (PVL 3827): 0.45

*Silesaurus opolensis* (ZPAL AbIII/363): 0.50

*Tanystropheus longobardicus* (Nosotti, 2007: table 5, MSNM BES SC 1018): 0.51

*Tanystropheus longobardicus* (Nosotti, 2007: table 5, MSNM BES SC 265): 0.50−0.51

*Tasmaniosaurus* (UTGD 54655): >0.41

*Trilophosauurs buettneri* (Spielmann et al., 2008: figs. 85b, 91a): 0.43

*Tropidosuchus romeri* (PVL 4601): 0.49

*Tropidosuchus romeri* (PVL 4606): 0.52

*Vancleavea campi* (Nesbitt et al., 2009: fig. 15): 0.63

*Youngina* (Smith & Evans, 1996: table 1, SAM-PK-K7710a): 0.54

*Youngina* (Smith & Evans, 1996: table 1, SAM-PK-K7710d): 0.50

Discretization by the cluster analysis: (1) 0.20−0.23; (2) 0.29−0.32; (3) 0.37−0.59; (4) 0.62−0.65. (5%=0.022).

Character 569:

*Aetosauroides scagliai* (PVL 2052): 0.69−0.74

*Amotosaurus* (SMNS 54783a): 0.58

*Azendohsaurus madagaskarensis* (UA 7-13-99-576): 0.68

*Azendohsaurus madagaskarensis* (UA 8-28-98-295): 0.65

*Chanaresuchus bonapartei* (Romer, 1972b: fig. 2): 0.42

*Dimorphodon* (Nesbitt, 2011: character 387): ≥0.85

*Euparkeria* (SAM-PK-7696): 0.63

*Euparkeria* (SAM-PK-8309): 0.56

GHG 7433MI: ca. 0.61

*Gracilisuchus* (PVL 4597): 0.79

*Herrerasaurus* (PVSJ 373): 0.61

*Heterodontosaurus* (SAM-PK-K1332): 0.56

*Lagerpeton chanarensis* (PULR 06): 0.17

*Lagerpeton chanarensis* (PVL 4619): 0.20

*Macrocnemus bessanii* (Peyer, 1937: 91, Alla Cascina specimen): 0.46

*Macrocnemus bessanii* (Peyer, 1937: 93, Besano III specimen): 0.75

*Macrocnemus bessanii* (Peyer, 1937: 94, Tre Fontane specimen 1936): 0.56−0.57

*Macrocnemus bessanii* (PIMUZ T4822): 0.46

*Macrocnemus bessanii* (PIMUZ T4355): 0.56

*Marasuchus lilloensis* (PVL 3870): 0.50

*Mesosuchus* (SAM-PK-7416): 0.41

*Noteosuchus* (AM 3591): 0.39

*Nundasuchus* (Nesbitt et al., 2014: table 1): 0.63

*Pamelaria dolichotracelos* (Sen, 2003: fig. 14i): 0.47

*Parasuchus hislopi* (ISI collection): 0.65

*Parasuchus hislopi* (ISI collection): 0.63

*Petrolacosaurus* (Reisz, 1981: fig. 24a): 0.56

*Prolacerta* (BP/1/2676): 0.72

*Proterosuchus fergusi* (SAM-PK-K140): 0.47

*Protorosaurus* (Gottmann-Quesada & Sander, 2009: appendix I, Nat. Kab. 191): 0.53

*Protorosaurus* (Gottmann-Quesada & Sander, 2009: appendix I, NHMW 1943I4): 0.47−0.48

*Protorosaurus* (Gottmann-Quesada & Sander, 2009: appendix I, NMK S180): 0.53

*Protorosaurus* (Gottmann-Quesada & Sander, 2009: appendix I, NMK S180): 0.54

*Protorosaurus* (Gottmann-Quesada & Sander, 2009: appendix I, PSM 7): 0.56

*Protorosaurus* (Gottmann-Quesada & Sander, 2009: appendix I, Simon/Bartholomäus specimen): 0.51

*Pseudochampsa ischigualastensis* (PVSJ 567): 0.32−0.33

*Rhynchosaurus articeps* (SHYMS 5): 0.42

*Rhynchosaurus articeps* (NHMUK PV R1238): 0.38

*Riojasuchus tenuisceps* (PVL 3827): 0.60

*Silesaurus opolensis* (ZPAL AbIII/363): >0.15

*Tanystropheus longobardicus* (Nosotti, 2007: table 5, MSNM BES SC 1018): 0.74

*Tanystropheus longobardicus* (Nosotti, 2007: table 5, MSNM BES SC 265): 0.66

*Trilophosauurs buettneri* (Spielmann et al., 2008: fig. 91a): 0.52

*Tropidosuchus romeri* (PVL 4601): 0.28

*Tropidosuchus romeri* (PVL 4606): 0.27

*Vancleavea campi* (Nesbitt et al., 2009: 836): 0.71−0.95

*Youngina* (Smith & Evans, 1996: table 1, SAM-PK-K7710b): 0.40

*Youngina* (Smith & Evans, 1996: table 1, SAM-PK-K7710d): 0.40

*Youngina* (Broom, 1921: fig. 20): 0.55

Discretization by the cluster analysis: (1) 0.17−0.21; (2) 0.27−0.33; (3) 0.38−0.42; (4) 0.46−0.79; (5) 0.93−0.97. (5%=0.04).

Character 571:

*Aetosauroides* (PVL 2073): 0.98

*Aetosauroides* (PVL 2052): 0.95

*Amotosaurus* (SMNS 54783a): 0.76

*Amotosaurus* (SMNS 90563): 0.83

*Azendohsaurus* (Nesbitt et al., in press: fig. 70): 0.83

*Chanaresuchus bonapartei* (Romer, 1972b: fig. 2f): 0.79

*Dimorphodon* (NHMUK PV R): 1.06

*Erythrosuchus* (BP/1/2096): 1.15

*Euparkeria* (GPIT 1681/1): 0.85

*Euparkeria* (SAM-PK-K8309): 0.91

GHG 7433MI: 0.84

*Gracilisuchus* (PVL 4597): 0.97

*Herrerasaurus* (PVSJ 373): 0.99

*Heterodontosaurus* (SAM-PK-K1332): 0.97

*Lagerpeton* (PULR 06): 0.52

*Lagerpeton* (PVL 4619): 0.53

*Macrocnemus* (PIMUZ T4822): 0.67

*Macrocnemus* (PIMUZ T4355): 0.73

*Macrocnemus* (PIMUZ T2472): 0.71

*Marasuchus* (PVL 3870): 0.90

*Marasuchus* (PVL 3871): 0.93

*Mesosuchus* (SAM-PK-7416): 0.63

*Noteosuchus* (AM 3591): 0.63

*Ornithosuchus* (NHMUK PV R2410): 1.00

*Pamelaria dolichotracelos* (Sen, 2003: fig. 14i): 0.79

*Parasuchus hislopi* (ISI collection): 0.83

*Parasuchus hislopi* (ISI collection): 0.84

*Petrolacosaurus* (Reisz, 1981: fig. 21): 0.56

*Proterosuchus fergusi* (SAM-PK-K140): 0.77

*Protorosaurus* (Gottmann-Quesada & Sander, 2009: appendix 1, IPB R 535): 0.61−0.67

*Protorosaurus* (Gottmann-Quesada & Sander, 2009: appendix 1, Nat. Kab. 191): 0.74

*Protorosaurus* (Gottmann-Quesada & Sander, 2009: appendix 1, NHMW 1974/1635): 0.63−0.68

*Protorosaurus* (Gottmann-Quesada & Sander, 2009: appendix 1, NMK S 180): 0.64

*Protorosaurus* (Gottmann-Quesada & Sander, 2009: appendix 1, NMK S182): 0.68

*Prolacerta* (Gow, 1975: fig. 24g): 0.75

*Rhynchosaurus articeps* (NHMUK PV R1238): 0.72

*Pseudochampsa* (PVSJ 567): 1.02

*Riojasuchus* (PVL 3827): 0.93

*Silesaurus* (ZPAL AbIII/363): 1.02

*Silesaurus* (ZPAL AbIII/1930): 0.97

*Tanystropheus* (Nosotti, 2007: table 7): 0.95

*Trilophosaurus* (Spielmann et al., 2008: fig. 91a): 0.71

*Tropidosuchus* (PVL 4601): 0.84

*Tropidosuchus* (PVL 4606): 0.82

*Youngina* (SAM-PK-K7710a): 0.60

*Youngina* (SAM-PK-K7710d): 0.61

Discretization by the cluster analysis: (1) 0.52−0.56; (2) 0.60−0.85; (3) 0.90−1.02; (4) 1.06−1.15. (5%=0.031).

Character 574:

*Aetosauroides scagliai* (PVL 2052): 1.00

*Amotosaurus* (SMNS 54783a): 1.15

*Azendohsaurus madagaskarensis* (UA 7-13-99-576): 1.07

*Azendohsaurus madagaskarensis* (UA 8-28-98-295): 1.08

*Boreopricea* (Benton & Allen, 1997: 944): 1.04

*Chanaresuchus bonapartei* (Romer, 1972b: fig. 2): 0.95

*Dimorphodon* (Padian, 1983: table 1, YPM 350): 0.98

*Erythrosuchus* (BP/1/2096): 0.94

*Euparkeria* (SAM-PK-7696): 1.06

*Euparkeria* (SAM-PK-8309): 0.91

GHG 7433MI: ca. 1.00

*Gracilisuchus* (PVL 4597): 0.95

*Herrerasaurus* (PVSJ 373): 0.87

*Heterodontosaurus* (SAM-PK-K1332): 0.90

*Lagerpeton chanarensis* (PULR 06): 1.12

*Lagerpeton chanarensis* (PVL 4619): 1.13

*Macrocnemus bessanii* (Peyer, 1937: 91, Alla Cascina specimen): 1.16

*Macrocnemus bessanii* (Peyer, 1937: 94, Tre Fontane specimen 1936): 1.17−1.19

*Macrocnemus bessanii* (PIMUZ T4822): 1.16

*Macrocnemus bessanii* (PIMUZ T4355): 1.13

*Marasuchus lilloensis* (PVL 3870): 0.99

*Marasuchus lilloensis* (PVL 3871): 0.89

*Mesosuchus* (SAM-PK-7416): 1.18

*Noteosuchus* (AM 3591): 1.22

*Pamelaria dolichotracelos* (Sen, 2003: fig. 14i): 1.17

*Parasuchus hislopi* (ISI collection): 1.04

*Parasuchus hislopi* (ISI collection): 1.06

*Petrolacosaurus* (Reisz, 1981: fig. 24a): 1.26

*Prolacerta* (BP/1/2676): 1.24

*Proterosuchus fergusi* (SAM-PK-K140): 1.11

*Protorosaurus* (Gottmann-Quesada & Sander, 2009: appendix I, IPB R 535): 1.23−1.33

*Protorosaurus* (Gottmann-Quesada & Sander, 2009: appendix I, Nat. Kab. 191): 1.00

*Protorosaurus* (Gottmann-Quesada & Sander, 2009: appendix I, NHMW 1943I4): 1.16−1.24

*Protorosaurus* (Gottmann-Quesada & Sander, 2009: appendix I, NMK S180): 1.16

*Protorosaurus* (Gottmann-Quesada & Sander, 2009: appendix I, PSM 7): 1.26

*Protorosaurus* (Gottmann-Quesada & Sander, 2009: appendix I, Simon/Bartholomäus specimen): 1.20

*Pseudochampsa ischigualastensis* (PVSJ 567): 0.85−0.92

*Rhynchosaurus articeps* (SHYMS 5): 1.13

*Rhynchosaurus articeps* (NHMUK PV R1238): 1.17

*Riojasuchus tenuisceps* (PVL 3827): 0.93

*Silesaurus opolensis* (ZPAL AbIII/361): 0.85

*Tanystropheus longobardicus* (Nosotti, 2007: table 5, MSNM BES SC 1018): 0.96

*Trilophosauurs buettneri* (Spielmann et al., 2008: fig. 91a): 1.22

*Tropidosuchus romeri* (PVL 4601): 0.96

*Tropidosuchus romeri* (PVL 4606): 0.99

*Youngina* (Smith & Evans, 1996: table 1, SAM-PK-K7710a): 1.18

*Youngina* (Smith & Evans, 1996: table 1, SAM-PK-K7710d): 1.20

*Youngina* (Broom, 1921: fig. 20): 1.28

Discretization by the cluster analysis: (1) 0.85−1.00; (2) 1.04−1.08; (3) 1.11−1.28; (4) 1.31−1.34. (5%=0.024).

Character 581:

*Amotosaurus* (SMNS 54783b): 0.83

*Azendohsaurus madagaskarensis* (UA 8-25-98-231): ca. 0.94

*Chanaresuchus bonapartei* (Romer, 1972b: fig. 2): 1.42

*Heterodontosaurus* (SAM-PK-K1332): ca. 1.00

*Lagerpeton chanarensis* (PVL 4619): 0.87

*Parasuchus hislopi* (ISI collection): 0.92

*Petrolacosaurus* (Peabody, 1952: fig. 9): 0.64

*Prolacerta* (BP/1/2676): 1.06

*Protorosaurus* (SMNS 55387, cast of Simon/Bartholomäus specimen): 0.77

*Protorosaurus* (NHMW 1943I4): 0.82

*Rhynchosaurus articeps* (SHYMS 5): 0.82

*Tanystropheus longobardicus* (Nosotti, 2007: table 5, MSNM BES SC 1018): 0.90

*Tanystropheus longobardicus* (PIMUZ T2817): 0.88

*Trilophosauurs buettneri* (Spielmann et al., 2008: fig. 91a): 0.73

*Youngina* (Smith & Evans, 1996: fig. 8a): 0.72

Discretization by the cluster analysis: (1) 0.64−0.77; (2) 0.82−0.83; (3) 0.87−1.44. (5%=0.04). Values between 0.87 and 1.44 were merged together because they were not informative discretized separately.

Character 583:

*Amotosaurus* (SMNS 54783a): 1.84

*Azendohsaurus madagaskarensis* (UA 8-25-98-231): 1.45

*Euparkeria* (SAM-PK-8309): 0.78

*Herrerasaurus* (PVSJ 373): 0.37

*Macrocnemus bessanii* (PIMUZ T4822): 1.73

*Parasuchus hislopi* (ISI collection): 1.42

*Petrolacosaurus* (Peabody, 1952: fig. 9): 1.91

*Prolacerta* (BP/1/2676): 1.87

*Protorosaurus* (SMNS 55387, cast of Simon/Bartholomäus specimen): 1.42

*Protorosaurus* (NHMW 1943I4): 1.86

*Protorosaurus* (Gottmann-Quesada & Sander, 2009: fig. 24, PSM 7): 1.44

*Tanystropheus longobardicus* (Nosotti, 2007: table 5, MSNM BES SC 265): 3.07

*Tanystropheus longobardicus* (Nosotti, 2007: table 5, MSNM BES SC 1018): 2.90

*Trilophosauurs buettneri* (Spielmann et al., 2008: fig. 91a): 1.74

*Youngina* (Smith & Evans, 1996, fig. 8a): 2.11

Discretization by the cluster analysis: (1) 0.30−0.85; (2) 1.37−3.07. (5%=0.135).
